# Photosynthetic responses to light levels in drought-tolerant novel peanut (*Arachis hypogaea* L) genotypes

**DOI:** 10.1038/s41598-025-10978-z

**Published:** 2025-07-11

**Authors:** Rajanna G. Adireddy, Saseendran S. Anapalli, Manisha Ojha, Naveen Puppala, Krishna N. Reddy

**Affiliations:** 1 Crop Production Systems Research Unit, USDA-ARS, Stoneville, MS 38776 USA; 2 ICAR-Indian Institute of Groundnut Research, Regional Research Station, Ananthapur, AP 515001 India; 3 Agricultural Science Center at Clovis, New Mexico State University, Clovis, NM 88101 USA; 4 Department of Plant and Environmental Sciences, New Mexico State University, Clovis, NM 88101 USA

**Keywords:** Chlorophyll fluorescence, Deficit irrigation, Electron transport rate, Peanut genotypes, Photosynthetic rate, Plant breeding, Environmental sciences

## Abstract

Drought is a significant abiotic stressor that reduces peanut production because it alters photosynthetic activity and impacts crop growth. Therefore, developing drought-tolerant peanut genotypes capable of maintaining higher photosynthetic rates (*A*) under stress is crucial. This study assessed changes in photosynthetic and chlorophyll fluorescence responses to light (photosynthetic photon flux density, PPFD) in newly bred drought-tolerant peanut genotypes. Ten genotypes [NM-3, NM-5, NM-6, NM-23, NM-69, NM-70, NM-74, NM-77, V-C, and C-76–16] were evaluated under full irrigation (FC_100_) and deficit irrigation (FC_50_) in a split-plot design with four replications in a greenhouse. Under high PPFD levels, genotype NM-5 with deficit irrigation exhibited significantly higher *A*, stomatal conductance (*gs*), quantum efficiency of photosystem II (ΦPSII), and electron transport rate (ETR) by 40–59%, 135–525%, 31–212%, and 31–102%, respectively, than check varieties (V-C and C-76–16) and other genotypes. The NM-74 and NM-77 genotypes also performed well under deficit irrigations but with slightly lower *A*, *gs*, ΦPSII, and ETR. Genotypes NM-5, NM-23, NM-74, and NM-77 exhibited significantly higher quantum efficiency of photosystem II (Fv’/Fm’) and photochemical quenching (qP) with higher light intensities in the daily cycle under deficit irrigation. The decline in ETR at the same PPFD levels in NM-3, NM 69, NM-70, and C-76–16 indicated photoinhibition or saturation of the photosynthetic apparatus compared to other genotypes. Concurrently, FC_100_ irrigation level minimizes photoinhibition, enhancing *A*, *gs*, ΦPSII, and ETR in the genotypes than FC_50_. Therefore, we conclude that NM-5, NM-74, and NM-77 genotypes can perform better under water deficit environments. As such, chlorophyll fluorescence parameters Fv’/Fm’ and qP can be considered for selective breeding to enhance photosynthetic efficiencies.

## Indroduction

Peanut (*Arachis hypogaea*) is an important oilseed crop, widely grown across continents, soils, and climates. Drought and temperature are two main abiotic factors causing severe losses in productivity and quality of peanut worldwide^1–3^. Drought impacts are more evident because about 90% of the world’s peanuts are cultivated in tropical and semi-arid regions^4,5^. According to a recent estimation, global peanut productivity incurred an annual loss of approximately 6 million tons^6^, costing about $520 million^7,8^ due to drought alone. Drought stress can be managed by developing drought-tolerant genotypes or by agronomic management that alleviates the adverse effects of drought. Developing peanut varieties through breeding approaches will provide a long-term solution to overcome drought limitations in peanut production^9–13^. When developing drought-tolerant genotypes, the focus should be on improving the physiological functions of the plant impacted by drought. Thus, possible genetic improvement strategies under drought-stressed environments should be a physio-genetic approach for optimizing yields^1,14–17^. Cultivar characteristics such as early stomatal closure, leaf area for maintaining optimum photosynthetic rates with high water use efficiency (WUE), and better root dynamics can be used to develop cultivars tolerant to drought^8,14,18,19^. Peanut plants respond to drought stress through changes in morphophysiological and agronomic characteristics that breeders can use to improve drought tolerance. Therefore, the development of drought-tolerant genotypes will be aided by a more profound understanding of how well genotypes survive in drought-stressed environments.

Understanding how various environmental factors influence plants’ photosynthetic rates (*A)* is essential for optimizing agricultural practices to improve crop resilience. One area of particular interest is the effect of light intensities the plants are exposed to in nature on the photosynthetic efficiency of different plant genotypes under varying water availability scenarios. Drought typically reduces stomatal conductance to conserve water, which in turn limits CO₂ uptake and reduces photosynthesis^20^. This effect can be exacerbated under high light conditions, where the reduced CO₂ availability can lead to increased production of reactive oxygen species and subsequent damage to the photosynthetic machinery^21^. Likewise, light is essential during photosynthesis to sustain energy transfer and assimilation processes by reducing adenosine triphosphate (ATP) and nicotinamide adenine dinucleotide phosphate (NADPH)^22^. Furthermore, drought stress exacerbates photoinhibition responses in photosystem II^23^. This can lead to the accumulation of reactive oxygen species (ROS) when excessive light energy is not sufficiently utilized in photosynthetic reactions^24^. This imbalance damages PSII, particularly D1 proteins, reducing photosynthetic efficiency and overall plant productivity^25,26^. Stomatal closure under drought exacerbates this by limiting CO₂ leads to accumulated ROS, which contributes to oxidative stress and inhibits normal photosynthetic processes^24^. To counter this effect, plants use non-photochemical quenching (NPQ) to protect against photoinhibition. NPQ replaces excess light energy as heat, preventing PSII damage^27,28^. Thus, NPQ mechanism contributes to alleviating drought-induced photoinhibition, maintaining higher photosynthetic efficiency and supporting drought tolerance. Excessive light can also cause photoinhibition and damage plants’ photosynthetic apparatus^29^. Conversely, low light conditions can limit the photosynthetic capacity, reducing growth and yield^30^. The interaction between light intensity and drought stress can affect various peanut genotypes differently. One physiological mechanism drought-tolerant genotypes employ is maintaining a higher photosynthesis and water use under drought stress than drought susceptible varieties^31^. Genotypes with enhanced *A* and more efficient water use during reproductive stages are more desirable for enhanced productivity^32,33^. Therefore, understanding how different peanut genotypes respond to drought and light is crucial for developing varieties with improved drought resilience. However, there is a lack of systematic quantification of photosynthetic responses to light in plants acclimated to various climate conditions^34^. Accurate quantification of morpho-physiological changes in peanut plants in response to light and drought can be challenging at the field scale, hindering selection of drought-tolerant genotypes based on the information^17^. Thus, studying physiological changes in peanut plants under drought stress in controlled greenhouse enclosures can provide fundamental information to explain growth variations related to environmental factors. However, chlorophyll fluorescence measurements like maximum efficiency of photosystem II, quantum yield, and photochemical and non-photochemical quenching offer real-time assessments of photosynthetic efficiency in plants. Therefore, this study investigates photosynthetic and chlorophyll fluorescence responses in peanut plants to light intensities between 0 to 2000 μ mol m^−2^ s^−1^ in ten newly bred peanut genotypes under full and deficit irrigation treatments.

## Results

### Photosynthetic rate (A)

**Fig. 1:**
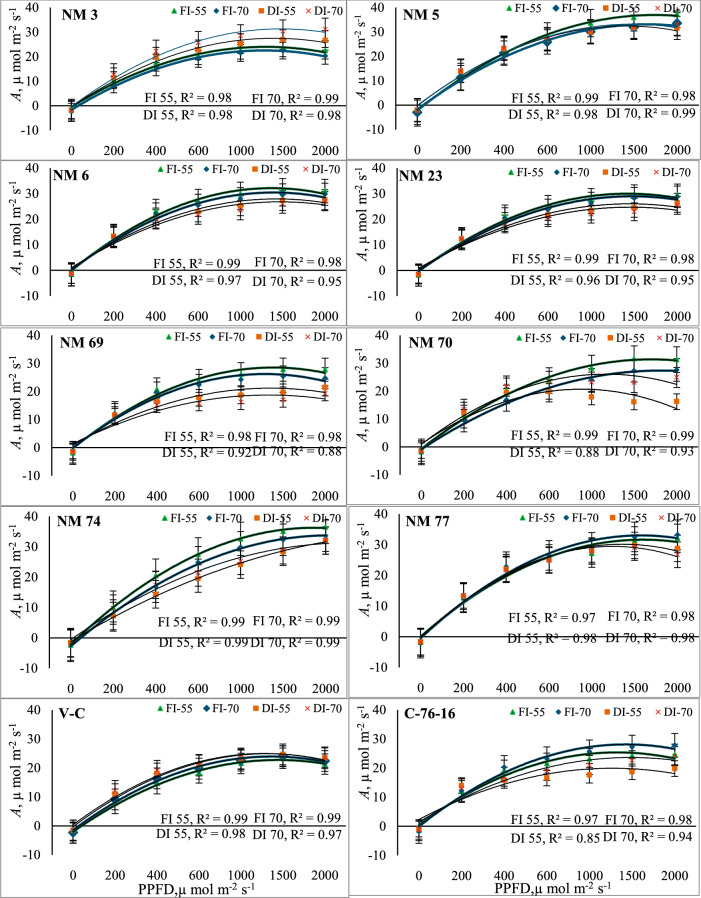
Photosynthetic rate (*A*) in response to variable light levels and moisture stress under different peanut genotypes. Note: Error bars in each polynomial line graph denote the standard error mean; FI-55 and FI-70, full irrigation at 55 and 70 days after sowing, respectively; DI-55 and DI-70, Deficit irrigation at 55 and 70 days after sowing, respectively.

At 55 and 70 DAP, across the 10 genotypes, *A* rates increased significantly with PPFD levels up to 1500 µmol m^2^ s⁻^1^ but declined with further increase. With NM-3, at 70 DAP, *A* increased significantly from −1.69 µmol CO₂ m⁻^2^ s⁻^1^ at 0 PPFD to 23.40 µmol CO₂ m⁻^2^ s⁻^1^ at 1500 µmol m^2^ s⁻^1^ (Fig. 1). This trend was consistent across other genotypes, NM-5, NM-6, NM-70, NM-74, and NM-77. However, NM-6, NM-74, and NM-77 had slightly lower *A* rates compared to NM-5 at higher PPFDs. Among irrigation levels, with FC_100_ rates, NM-5 exhibited an increase in *A* rate significantly from −3.01 µmol CO₂ m⁻^2^ s⁻^1^ at 0 PPFD to 29.88 µmol CO₂ m⁻^2^ s⁻^1^ at 1000 PPFD, reaching a peak of 31.28 µmol CO₂ m⁻^2^ s⁻^1^ at 1500 PPFD before slightly declining at 2000 PPFD. At 70 DAP, NM-5 reached its peak *A* rate of 33.60 µmol CO₂ m⁻^2^ s⁻^1^ at 2000 PPFD. Similarly, NM-74 and NM-77 significantly increased *A* rate from 0 to 2000 PPFD levels, slightly lower than NM-5. Genotype NM-74 reached a maximum *A* of 33.65 µmol CO₂ m⁻^2^ s⁻^1^ at 2000 PPFD at 70 DAP, while C-76–16 peaked at 27.83 µmol CO₂ m⁻^2^ s⁻^1^ at 2000 PPFD at 70 DAP (Fig. 1). Similarly, under deficit irrigation treatment (FC_50_), genotypes NM-5 and NM-74 were more efficient in enhancing *A* significantly by exhibiting > 30 µmol CO₂ m⁻^2^ s⁻^1^ *A* rate at 2000 PPFD level than other genotypes. NM-5 under deficit irrigation exhibited an increase in *A* by 40–59% over check varieties and other genotypes. However, all the genotypes exhibited significantly lower *A* rates under FC_50_ than FC100, except NM-3, which has higher *A* rates under drought than well-watered conditions. Among interactions, under FC_100_, all genotypes exhibited significantly higher *A* than under FC_50_ excepting for NM-3 and V-C genotypes.

### Transpiration rate (Tr)

Transpiration rates increased with higher PPFD levels for most genotypes, though the extent of the increase varied among genotypes, irrigation levels, and measurement days. Among genotypes, the NM-5 genotype exhibited significantly higher Tr than other peanut genotypes, starting at 6.09 mmol H_2_O m⁻^2^ s⁻^1^ at 0 PPFD and reaching 16.48 mmol H_2_O m⁻^2^ s⁻^1^ at 2000 PPFD at 55 DAP and 16.25 mmol H_2_O m⁻^2^ s⁻^1^ at 70 DAP (Table 1). Under NM-70, the Tr rates at 9.94 mmol H_2_O m⁻^2^ s⁻^1^ at 55 DAP and 7.07 mmol H_2_O m⁻^2^ s⁻^1^ at 70 DAP at 0 PPFD, with 15.90 mmol H_2_O m⁻^2^ s⁻^1^ at 2000 PPFD at 55 DAP and 14.32 mmol H_2_O m⁻^2^ s⁻^1^ at 70 DAP. All these two genotypes exhibited significantly higher Tr rates than the check genotypes of V-C and C-76–16. Interestingly, NM-3, NM-6, and NM-23 followed similar patterns but exhibited slightly lower rates compared to NM-5, with maximum values of 10.29, 11.38, and 11.68 mmol H_2_O m⁻^2^ s⁻^1^, respectively, at 2000 PPFD at 70 DAP. NM-69 had lower Tr rates than other genotypes. Among checks and other genotypes, C76-16 showed significantly lower Tr rates between 4.62 mmol H_2_O m⁻^2^ s⁻^1^ at 55 DAP and 6.00 mmol H_2_O m⁻^2^ s⁻^1^ at 70 DAP at 0 PPFD and 7.67 mmol H_2_O m⁻^2^ s⁻^1^ at 2000 PPFD at 55 DAP and 9.71 mmol H_2_O m⁻^2^ s⁻^1^ at 70 DAP.

**Table 1:** Transpiration rate (Tr) and maximum quantum efficiency of photosystem II (Fv’/Fm) in different peanut genotypes under variable light intensities.

Genotype	PPFD level (mol m⁻^2^ s⁻^1^)	Tr (mmol H₂O m⁻^2^ s⁻^1^)				Fv’/Fm’			
		Full irrigation		Deficit irrigation		Full irrigation		Deficit irrigation	
		55 day	70 day	55 day	70 day	55 day	70 day	55 day	70 day
NM 3	0	3.83f.	3.17f.	3.66 g	4.66f.	-	-	-	-
	200	4.81e	4.18ef	6.29f.	6.98e	0.62a	0.65a	0.70a	0.73a
	400	6.02d	5.57de	7.68e	8.57de	0.59b	0.62ab	0.67abc	0.71ab
	600	7.12c	7.01 cd	8.69d	9.72 cd	0.55 cd	0.59bc	0.64 cd	0.68ab
	1000	8.34b	8.37bc	9.64c	10.70bc	0.53d	0.56c	0.64d	0.67b
	1500	9.46a	9.55ab	10.90b	11.79ab	0.54d	0.57c	0.65bcd	0.69ab
	2000	10.29a	10.31a	12.39a	12.90a	0.58bc	0.60abc	0.68ab	0.71ab
NM 5	0	6.09 g	6.19f.	11.03e	13.62b	-	-	-	-
	200	8.31f.	8.16e	12.36d	14.51 cd	0.76a	0.74a	0.73a	0.75a
	400	10.95e	10.74d	13.48c	15.23bcd	0.74ab	0.72a	0.69b	0.71ab
	600	12.75d	12.56c	14.68b	15.91bc	0.71bc	0.70a	0.65c	0.68b
	1000	13.83c	13.75bc	15.76a	16.85ab	0.69c	0.69a	0.62 cd	0.66b
	1500	15.02b	14.92ab	16.66a	17.78a	0.69c	0.70a	0.62d	0.66b
	2000	16.48a	16.25a	16.65a	17.92a	0.71c	0.72a	0.60d	0.66b
NM 6	0	7.16f.	6.50e	7.82f.	6.11d	-	-	-	-
	200	8.12e	7.62de	8.64ef	6.89 cd	0.72a	0.68a	0.75a	0.76a
	400	9.04d	8.64 cd	9.33de	7.48 cd	0.69ab	0.64ab	0.70b	0.71ab
	600	9.71 cd	9.30bcd	10.01 cd	7.73 cd	0.65 cd	0.60bc	0.67c	0.67bc
	1000	10.36bc	9.94abc	10.91bc	8.48bc	0.63d	0.58c	0.66c	0.64c
	1500	10.93ab	10.64ab	11.64ab	9.63ab	0.65d	0.60bc	0.67c	0.65c
	2000	11.38a	11.41a	12.06a	10.50a	0.68bc	0.64ab	0.67c	0.67bc
NM 23	0	7.42f.	7.70e	5.10e	4.33 cd	-	-	-	-
	200	8.27ef	8.55de	4.86e	3.72d	0.75a	0.75a	0.75a	0.75a
	400	9.05de	9.39cde	5.35de	4.12dcd	0.73ab	0.72a	0.73a	0.73a
	600	9.65 cd	10.07bcd	6.09 cd	4.90bcd	0.71b	0.71a	0.68b	0.67b
	1000	10.33bc	10.91abc	6.85bc	5.68bc	0.71b	0.71a	0.64c	0.62b
	1500	10.99ab	11.69ab	7.44b	6.25ab	0.73ab	0.73a	0.65c	0.62b
	2000	11.68a	12.45a	8.87a	7.93a	0.75a	0.75a	0.68d	0.65b
NM 69	0	6.98d	5.59c	3.58c	2.45a	-	-	-	-
	200	7.59 cd	6.26bc	3.66bc	2.35a	0.75a	0.75a	0.76a	0.77a
	400	7.93bc	6.67abc	3.80bc	2.42a	0.71b	0.71ab	0.72b	0.72a
	600	8.29bc	7.07abc	3.82bc	2.33a	0.67 cd	0.67bc	0.66c	0.65b
	1000	8.80ab	7.49ab	4.22bc	2.69a	0.65d	0.64c	0.61d	0.59c
	1500	9.41a	8.11a	4.53ab	2.91a	0.66d	0.64c	0.61d	0.59c
	2000	9.68a	8.37a	5.18a	3.68a	0.69bc	0.68bc	0.67c	0.66b
NM 70	0	9.94f.	7.07f.	5.62a	7.67b	-	-	-	-
	200	10.88e	7.99ef	5.78a	8.20ab	0.73a	0.72ab	0.74a	0.74a
	400	11.76e	9.20de	5.77a	8.48ab	0.69bc	0.67b	0.71b	0.71ab
	600	12.84d	10.62 cd	5.53a	8.75ab	0.68c	0.67b	0.66c	0.67bc
	1000	13.79c	11.82bc	5.04a	8.76ab	0.69bc	0.69ab	0.59d	0.63c
	1500	14.76b	12.93ab	5.00a	9.08ab	0.72ab	0.71ab	0.59d	0.63c
	2000	15.90a	14.32a	5.27a	9.61a	0.74a	0.74a	0.67c	0.68b
NM 74	0	5.27 g	3.78e	2.20f.	4.24f.	-	-	-	-
	200	6.65f.	4.68de	3.00f.	4.93ef	0.74a	0.73a	0.72a	0.74a
	400	8.32e	6.28d	4.23e	6.09de	0.71b	0.68ab	0.69b	0.71a
	600	10.41d	9.11c	5.32d	7.05 cd	0.69bc	0.67b	0.67bc	0.70a
	1000	12.21c	11.54b	6.68c	8.19bc	0.68c	0.64b	0.66c	0.69a
	1500	13.77b	13.53a	8.30b	9.32ab	0.68c	0.64b	0.67bc	0.71a
	2000	15.15a	15.24a	10.12a	10.53a	0.69bc	0.65b	0.69b	0.72a
NM 77	0	8.34e	8.79e	5.16f.	5.47d	-	-	-	-
	200	9.00e	9.86de	5.71ef	6.14d	0.72a	0.73a	0.76a	0.76a
	400	9.99d	11.05 cd	6.63de	7.25 cd	0.70a	0.71ab	0.73a	0.74ab
	600	10.75 cd	11.95bc	7.38 cd	8.19bc	0.66b	0.67b	0.69b	0.71ab
	1000	11.52bc	12.94ab	8.20bc	9.20ab	0.64b	0.66b	0.66c	0.70b
	1500	12.16b	13.70ab	8.77ab	9.96ab	0.63b	0.66b	0.65c	0.69b
	2000	13.11a	14.65a	9.15a	10.50a	0.65b	0.67b	0.66bc	0.69b
V-C	0	11.18e	9.69d	7.33d	6.37d	-	-	-	-
	200	11.59de	10.19 cd	7.65d	6.74 cd	0.67a	0.69a	0.73a	0.74a
	400	12.06cde	10.65bcd	7.99 cd	7.22 cd	0.64b	0.66ab	0.71ab	0.71ab
	600	12.29 cd	11.00bcd	8.25bcd	7.73bcd	0.62bc	0.64b	0.69b	0.69ab
	1000	12.78bc	11.54abc	8.66abc	8.49abc	0.60c	0.62b	0.68b	0.68b
	1500	13.45ab	12.21ab	9.06ab	9.08ab	0.61c	0.63b	0.69b	0.70ab
	2000	14.16a	12.88a	9.45a	9.59a	0.60c	0.64b	0.69b	0.72ab
C-76–16	0	4.62e	6.00d	4.10a	3.78a	-	-	-	-
	200	5.20de	6.59 cd	2.88b	2.97a	0.75a	0.75a	0.68a	0.71a
	400	5.70 cd	7.19bcd	2.15b	2.66a	0.70b	0.71ab	0.58b	0.64b
	600	6.04bcd	7.72bcd	2.08b	2.89a	0.66c	0.67b	0.54c	0.60bc
	1000	6.31bc	8.19abc	2.18b	3.26a	0.64c	0.65b	0.53c	0.57c
	1500	6.82ab	8.93ab	2.15b	3.58a	0.66c	0.67b	0.54c	0.57c
	2000	7.67a	9.71a	2.23b	3.99a	0.69b	0.71b	0.56bc	0.59c

Means in columns with the same letter or combination of letters are not significantly different.

Among irrigation levels, the results indicated a consistent trend where Tr rates increase with higher PPFD. Generally, full irrigation resulted in significantly higher Tr rates than deficit irrigation across all genotypes and PPFD levels. Likewise, the difference in Tr rates became more pronounced at higher PPFD levels. The NM-3, NM-5, and NM-6 genotypes exhibited higher Tr rates under deficit irrigation applied treatments than full irrigation across all PPFD levels. Among all genotypes, NM-5 exhibited significantly higher Tr rates at 16.65 mmol H_2_O m⁻^2^ s⁻^1^ (55 DAP) and 17.92 mmol H_2_O m⁻^2^ s⁻^1^ (70 DAP) under deficit irrigation at the highest PPFD level (Table 1). The percent increase in Tr under the NM-5 genotype with deficit irrigation was 58–176% over check varieties and other genotypes. Among interactions, under FC_100,_ all genotypes exhibited significantly higher Tr than under FC_50_ excepting NM-3 and NM-5 genotypes.

### Stomatal conductance (g_s_)

**Fig. 2:**
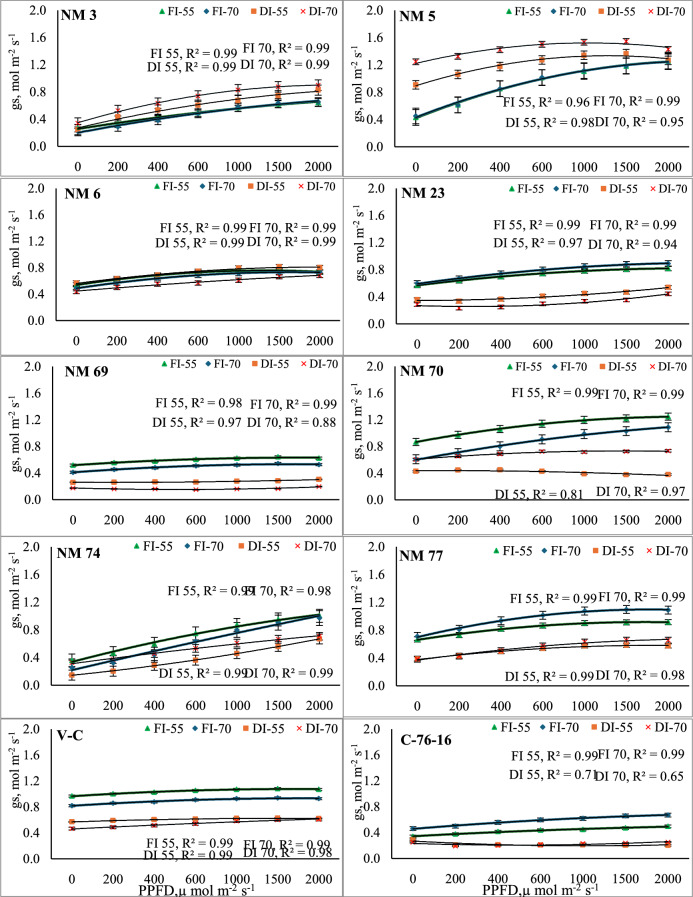
Stomatal conductivity (gs) in response to variable light levels and moisture stress under different peanut genotypes. Note: Error bars in each polynomial line graph denote the standard error mean; FI-55 and FI-70, full irrigation at 55 and 70 days after sowing, respectively; DI-55 and DI-70, Deficit irrigation at 55 and 70 days after sowing, respectively.

Substantial variations in *g*_*s*_ among genotypes were observed with increasing PPFD levels. Under FC_100_, NM-5 exhibited a more pronounced increase (P < 0.01) in *gs* with increasing PPFD at 55 DAP, from 0.43 mol m⁻2 s⁻1 at 0 PPFD to 1.26 mol m⁻2 s⁻1 at 2000 PPFD. At 70 DAP, the *gs* were slightly higher at each PPFD level, ending in 1.25 mol m⁻2 s⁻1 at 2000 PPFD (Fig. 2). Similarly, NM-70 also recorded higher *gs* values, slightly lower than NM-5. Thus, in NM-70, *gs* increased significantly from 0.87 mol m⁻2 s⁻1 at 0 PPFD to 1.25 mol m⁻2 s⁻1 at 2000 PPFD at 55 DAP and from 0.61 mol m⁻2 s⁻1 to 1.09 mol m⁻2 s⁻1 at 70 DAP. Similarly, NM-74 showed a slower rate of increase in *gs* than NM-5, NM-70, and NM-74 genotypes. These genotypes exhibited significantly higher *gs* rates than both the check varieties. Other genotypes like NM-3, NM-6, NM-23, and NM-69 exhibited the lowest *gs* values across all PPFD and irrigation levels. Among check varieties, the V-C genotype showed relatively higher *gs* across all PPFD levels than C-76–16.

Among irrigation levels, deficit irrigation (FC_50_) treatment exhibited lower *gs* rates across all genotypes under all PPFD levels and growth stages. However, genotypes NM-3, NM-5, and NM-6 exhibited significantly higher *gs* rates under deficit irrigation treatment than under FC_100_ irrigation treatment than other genotypes. Interestingly, the NM-5 maintained equal stomatal conductance by exhibiting significantly higher *gs* under both irrigation levels across all PPFD levels. NM-5 exhibited 535% higher stomatal conductance than the check genotype C-76–16 (Fig. 2). Among checks, V-C exhibited 210% higher *gs* rates compared to C-76–16. Among interactions, irrigation under FC_100,_ all genotypes exhibited significantly higher Tr than under FC_50_ except for NM-3, NM-5 and NM-6 genotypes.

### Quantum yield of photosystem II (ΦPSII)

**Fig. 3:**
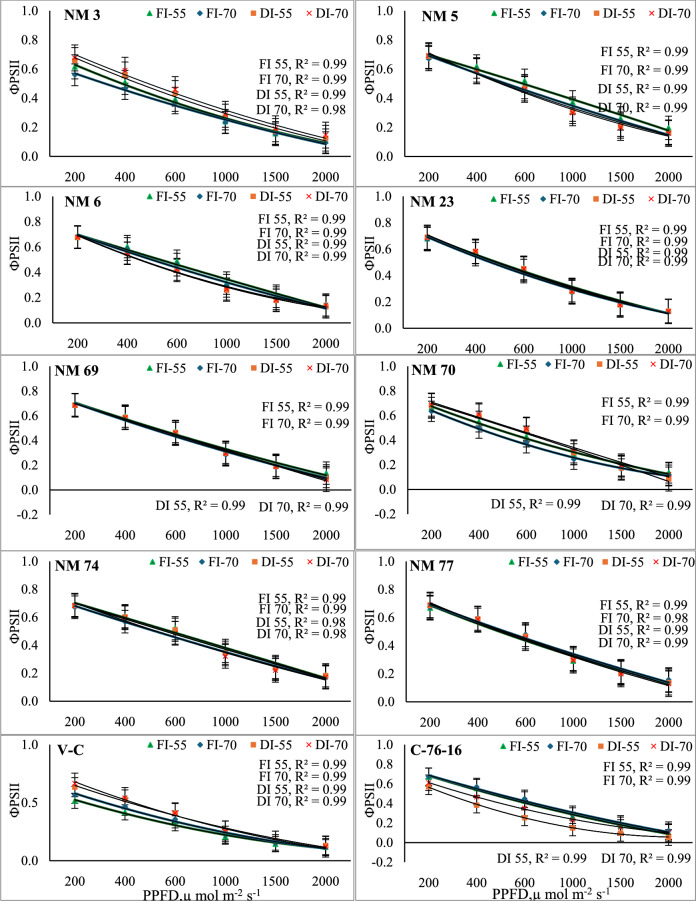
Quantum yield of photosystem II (ФPSII) in response to variable light levels and moisture stress under different peanut genotypes. Note: Error bars in each polynomial line graph denote the standard error mean; FI-55 and FI-70, full irrigation at 55 and 70 days after sowing, respectively; DI-55 and DI-70, Deficit irrigation at 55 and 70 days after sowing, respectively.

All the genotypes under both irrigation levels exhibited a decrease in *ΦPSII* with increasing PPFD levels. Though all the genotypes exhibited a decline in *ΦPSII* with increased PPFD levels, genotypes like NM-5, NM-74, and NM-77 exhibited significantly higher ΦPSII than other peanut genotypes. These three genotypes maintained a ΦPSII of 0.67–0.69 µmol m2 s⁻1 at 200 PPFD to 0.16–0.17 µmol m2 s⁻1 at 2000 PPFD (Fig. 3). At the same time, the NM-3 exhibited the lowest ΦPSII over all other studied genotypes. Genotypes like NM-6, NM-23, NM-69, and NM-70 exhibited similar ΦPSII values compared to V-C and C-76–16 check varieties. Similarly, both the check varieties of V-C and C-76–16 exhibited lower quantum efficiency values than other genotypes. Among irrigation levels, under FC_50_, NM-3, NM-6, NM-74, NM-77, and V-C exhibited higher ΦPSII over other genotypes. Interestingly, NM-5 and NM-70 exhibited lower ΦPSII under FC_100_ than in FC_50_. Likewise, NM-5 with FC_50_ exhibited a significant increase in ΦPSII by 31–212% at higher light intensity of 1500–2000 PPFD over check varieties and other genotypes.

### Electron transport rate (ETR)

**Fig. 4:**
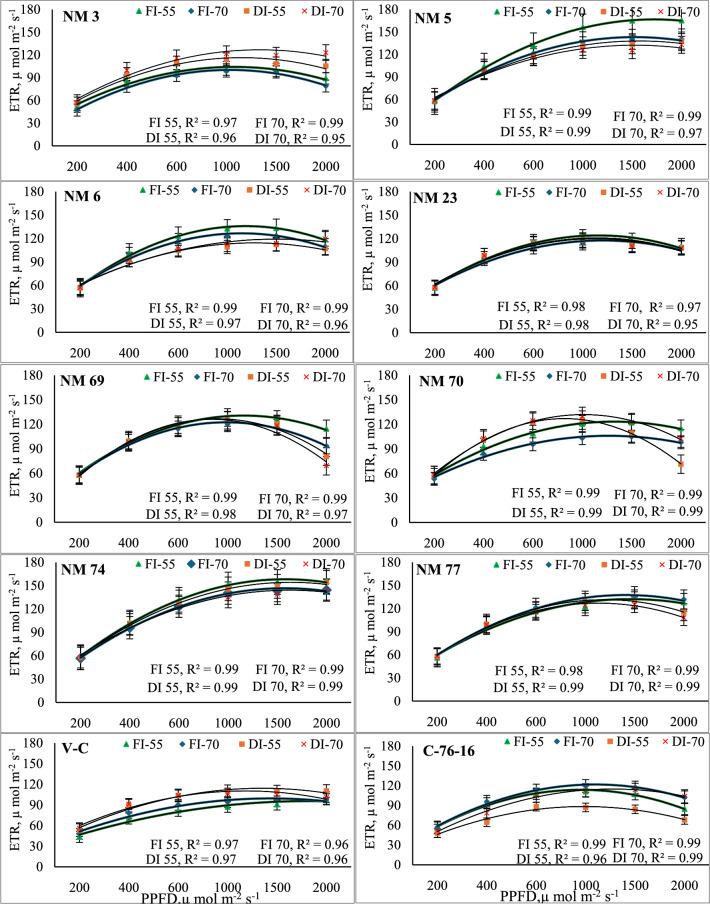
Electron transport rate (ETR) in response to variable light levels and moisture stress under different peanut genotypes. Note: Error bars in each polynomial line graph denote the standard error mean; FI-55 and FI-70, full irrigation at 55 and 70 days after sowing, respectively; DI-55 and DI-70, Deficit irrigation at 55 and 70 days after sowing, respectively.

The electron transport rate comparison between FC_50_ and FC_100_ across various genotypes and PPFD levels revealed distinct trends and responses at 55 and 70 DAP. Generally, ETR was significantly higher under FC_100_ conditions across most genotypes and PPFD levels, particularly at 55 DAP. At a PPFD of 1000 µmol m⁻^2^ s⁻^1^, NM-5 exhibited an ETR of 156.4 µmol electrons m⁻^2^ s⁻^1^ under FC_100_ at 55 DAP compared to 131.2 µmol electrons m⁻^2^ s⁻^1^ under deficit irrigation (FC_50_). As PPFD increased, ETR was observed to increase for both irrigation treatments. Among genotypes, NM-6 showed a significant increase in ETR from 57.1 µmol electrons m⁻^2^ s⁻^1^ at 200 µmol m⁻^2^ s⁻^1^ to 132.5 µmol electrons m⁻^2^ s⁻^1^ at 1000 µmol m⁻^2^ s⁻^1^ under FC_100_ at 55 DAP (Fig. 4). In contrast, under FC_50_, ETR values for NM-6 at the same PPFD levels were lower but still showed an increase, from 57.0 to 109.2 µmol electrons m⁻^2^ s⁻^1^. Likewise, NM-5 and NM-74 genotypes displayed remarkable tolerance under deficit irrigation by maintaining higher ETR values than other genotypes. Likewise, NM-5 achieved an ETR of 165.2 µmol electrons m⁻^2^ s⁻^1^ in 2000 µmol m⁻^2^ s⁻^1^ with deficit irrigation (FC_50_) at 70 DAP. Likewise, NM-5 with deficit irrigation exhibited a significant increase in ETR by 31–102% over check varieties and other genotypes. Similarly, peanut growth extending from 55 to 70 DAP generally increased ETR, particularly for deficit irrigation treatment. Genotype NM-3, ETR under deficit irrigation in 2000 µmol m⁻^2^ s⁻^1^ increased from 105.4 µmol electrons m⁻^2^ s⁻^1^ at 55 DAP to 123.1 µmol electrons m⁻^2^ s⁻^1^ at 70 DAP. Conversely, some genotypes showed a decrease in ETR with prolonged deficit irrigation. In genotype NM-69, ETR in 2000 µmol m⁻^2^ s⁻^1^ dropped from 114.5 µmol electrons m⁻^2^ s⁻^1^ at 55 DAP to 93.4 µmol electrons m⁻^2^ s⁻^1^ at 70 DAP (Fig. 4). Genotypes such as NM-70 and NM-69, experienced significant declines in ETR under deficit irrigation at higher PPFD levels. Among checks, C-76–16 produced lowest ETR values among all treatments investigated, particularly under the deficit irrigation treatment.

### Chlorophyll fluorescence

Chlorophyll fluorescence parameters such as Fv’/Fm’ (maximum quantum efficiency of PSII) of different genotypes under varying PPFD levels and irrigation conditions were determined. Generally, Fv’/Fm’ decreased with increasing PPFD levels across all genotypes and irrigation levels. For NM-3 under FC_100_, a notable decline (P < 0.01) in Fv’/Fm’ was observed with increasing PPFD levels, with values ranging from 0.62 at 200 µmol m⁻2 s⁻1 to 0.53 at 1000 µmol m⁻2 s⁻1 at 55 DAP (Table 1). In contrast, NM-5 displayed significantly higher Fv’/Fm,’ with maximum values of 0.76 at 200 µmol m⁻2 s⁻1. Similarly, NM-23 consistently performed with Fv’/Fm’ at 0.75 across both irrigation treatments at 200 µmol m⁻2 s⁻1. In contrast, NM-69, NM-70, and C-76–16 demonstrated a decline in Fv’/Fm’ at higher PPFD levels, particularly under deficit irrigation (FC_50_) compared to FC_100_. Genotypes NM-6, NM-69, NM-74, and NM-77 exhibited a similar pattern, maintaining significantly high Fv’/Fm’ under both irrigation levels at all PPFD levels with values greater than check genotype C76-16 (Table 1). Among all genotypes, C-76–16 exhibited significantly lowesr Fv’/Fm’ values, particularly under deficit irrigation, reaching a minimum of 0.53 at 1000 µmol m⁻2 s⁻1.

### Photochemical quenching (qP)and Non-photochemical quenching (qN)

Generally, qP decreased with increasing PPFD in genotypes and irrigation levels. NM-3 exhibited qP from 1.02 at 200 µmol m⁻^2^ s⁻^1^ to 0.19 at 2000 µmol m⁻^2^ s⁻^1^ under FC_100_ at 55 DAP. While NM-5, NM-23, NM-74, and NM-77 exhibited slightly lower but relatively stable qP values (~ 0.90) at 200 µmol m⁻^2^ s⁻^1^ 55 DAP (Table 2). This pattern continued at 70 DAP, where qP values remained consistently higher than NM-3, NM-6, and C-76–16. Similarly, NM-6 under full irrigation exhibited significantly higher qP values (0.96) at 200 µmol m⁻^2^ s⁻^1^ but declined at higher PPFD levels. C-76–16 displayed the lowest qP values across most treatments, particularly under FC_50_, where values dipped to 0.09 with PPFD at 2000 µmol m⁻^2^ s⁻^1^.

**Table 2:** Photochemical quenching (qP) and non-photochemical quenching (qN) in different peanut genotypes under variable light intensities.

Genotype	PPFD level (mol m⁻^2^ s⁻^1^)	qP				qN			
		Full irrigation		Deficit irrigation		Full irrigation		Deficit irrigation	
		55 day	70 day	55 day	70 day	55 day	70 day	55 day	70 day
NM 3	0	-	-	-	-	-	-	-	-
	200	1.02a	0.89a	0.95a	0.92a	0.00c	0.00d	0.00d	0.08bc
	400	0.89b	0.77b	0.84b	0.83a	0.03b	0.11 cd	0.15bc	0.20ab
	600	0.74c	0.65c	0.68c	0.67b	0.27a	0.30abc	0.30a	0.35a
	1000	0.46d	0.43d	0.43d	0.43c	0.37a	0.41a	0.33a	0.38a
	1500	0.29e	0.27e	0.26e	0.27d	0.27a	0.37ab	0.24ab	0.31a
	2000	0.19f.	0.16f.	0.19f.	0.21d	0.07b	0.20bc	0.11c	0.20ab
NM 5	0	-	-	-	-	-	-	-	-
	200	0.90a	0.91a	0.94a	0.93a	0.00d	0.11bc	0.00d	0.00c
	400	0.84b	0.83b	0.85b	0.83b	0.11c	0.22ab	0.20c	0.14bc
	600	0.72c	0.69c	0.72c	0.68c	0.24b	0.33a	0.38b	0.30ab
	1000	0.54d	0.47d	0.50d	0.46d	0.36a	0.39a	0.49a	0.34a
	1500	0.38e	0.32e	0.34e	0.30e	0.36a	0.35a	0.51a	0.31ab
	2000	0.28f.	0.24f.	0.27f.	0.24e	0.30ab	0.23ab	0.54a	0.31ab
NM 6	0	-	-	-	-	-	-	-	-
	200	0.96a	1.01a	0.91a	0.89a	0.00d	0.00b	0.00c	0.00c
	400	0.89b	0.93b	0.78b	0.78b	0.10bc	0.12b	0.23b	0.27b
	600	0.75c	0.77c	0.62c	0.63c	0.29a	0.32a	0.39a	0.45ab
	1000	0.50d	0.51d	0.40d	0.42d	0.39a	0.40a	0.44a	0.53a
	1500	0.33e	0.33e	0.27e	0.28e	0.33a	0.32a	0.40a	0.51a
	2000	0.21f.	0.20f.	0.19f.	0.21e	0.15b	0.12b	0.37a	0.46ab
NM 23	0	-	-	-	-	-	-	-	-
	200	0.91a	0.90a	0.91a	0.91a	0.04c	0.00c	0.00e	0.00b
	400	0.80b	0.78b	0.80b	0.80b	0.17ab	0.16ab	0.14d	0.13b
	600	0.64c	0.61c	0.66c	0.67c	0.25ab	0.24a	0.37bc	0.41a
	1000	0.41d	0.38d	0.44d	0.45d	0.26a	0.22a	0.49a	0.57a
	1500	0.26e	0.24e	0.28e	0.28e	0.15b	0.10ab	0.47ab	0.57a
	2000	0.17f.	0.17f.	0.19f.	0.20e	0.01c	0.00c	0.36c	0.48a
NM 69	0	-	-	-	-	-	-	-	-
	200	0.92a	0.92a	0.90a	0.89a	0.02d	0.04c	0.00d	0.00c
	400	0.84b	0.82b	0.82b	0.81a	0.21c	0.25b	0.20c	0.19b
	600	0.70c	0.68c	0.71c	0.71b	0.40ab	0.43ab	0.46b	0.48a
	1000	0.47d	0.45d	0.49d	0.50c	0.48a	0.52a	0.60a	0.64a
	1500	0.30e	0.29e	0.32e	0.32d	0.45a	0.50a	0.60a	0.66a
	2000	0.19f.	0.16f.	0.14f.	0.13e	0.31bc	0.38ab	0.43b	0.47a
NM 70	0	-	-	-	-	-	-	-	-
	200	0.90a	0.88a	0.91a	0.93a	0.00c	0.06 cd	0.00d	0.00d
	400	0.79b	0.73b	0.84b	0.86a	0.22a	0.30ab	0.13c	0.12 cd
	600	0.63c	0.56c	0.74c	0.74b	0.29a	0.33a	0.38b	0.33ab
	1000	0.41d	0.35d	0.51d	0.50c	0.23a	0.25abc	0.55a	0.43a
	1500	0.27e	0.23e	0.29e	0.31d	0.11b	0.11bcd	0.53a	0.38ab
	2000	0.19f.	0.16f.	0.13f.	0.17e	0.00c	0.00d	0.31b	0.22bc
NM 74	0	-	-	-	-	-	-	-	-
	200	0.93a	0.92a	0.94a	0.93a	0.00c	0.03b	0.00c	0.00c
	400	0.85b	0.84b	0.87b	0.84a	0.16b	0.27a	0.03bc	0.10abc
	600	0.75c	0.73c	0.76c	0.70b	0.25ab	0.33a	0.15a	0.19ab
	1000	0.53d	0.52d	0.52d	0.47c	0.33a	0.42a	0.19a	0.22a
	1500	0.36e	0.35e	0.35e	0.31d	0.32a	0.42a	0.11ab	0.14ab
	2000	0.27f.	0.26f.	0.27f.	0.24d	0.27a	0.37a	0.01bc	0.05abc
NM 77	0	-	-	-	-	-	-	-	-
	200	0.93a	0.92a	0.91a	0.91a	0.00c	0.00b	0.00c	0.00c
	400	0.84b	0.84b	0.81b	0.79b	0.00c	0.00b	0.00c	0.02bc
	600	0.70c	0.71c	0.68c	0.64c	0.04b	0.05a	0.20b	0.17ab
	1000	0.46d	0.47d	0.46d	0.43d	0.16a	0.14a	0.34a	0.25a
	1500	0.33e	0.33e	0.32e	0.29e	0.18a	0.13a	0.37a	0.25a
	2000	0.23f.	0.23f.	0.21f.	0.19f.	0.09ab	0.03a	0.31a	0.27a
V-C	0	-	-	-	-	-	-	-	-
	200	0.76a	0.81a	0.86a	0.90a	0.25c	0.17bc	0.04c	0.03b
	400	0.64b	0.69b	0.74b	0.76b	0.37b	0.32ab	0.16b	0.18ab
	600	0.52c	0.55c	0.59c	0.60c	0.47ab	0.43a	0.26ab	0.30a
	1000	0.34d	0.36d	0.38d	0.37d	0.52a	0.47a	0.29a	0.33a
	1500	0.24e	0.24e	0.25e	0.24e	0.50a	0.43a	0.25ab	0.26a
	2000	0.20e	0.19f.	0.19f.	0.17e	0.51a	0.40a	0.22ab	0.17ab
C-76–16	0	-	-	-	-	-	-	-	-
	200	0.90a	0.90a	0.84a	0.86a	0.00c	0.02c	0.38c	0.25c
	400	0.79b	0.80b	0.65b	0.71b	0.26b	0.26b	0.63ab	0.46b
	600	0.65c	0.67c	0.45c	0.56c	0.44a	0.42ab	0.72a	0.59ab
	1000	0.41d	0.44d	0.27d	0.38d	0.51a	0.47a	0.74a	0.67a
	1500	0.26e	0.27e	0.17e	0.26e	0.44a	0.38ab	0.72a	0.67a
	2000	0.14f.	0.17f.	0.09f.	0.17e	0.30b	0.24b	0.67ab	0.64ab

Means in columns with the same letter or combination of letters are not significantly different.

Genotypes NM-3, NM-5, NM-6, NM-70, NM-74, and NM-77 exhibited variable qN values, with zero values at lower PPFD levels under both irrigation treatments. However, at 600 µmol m⁻^2^ s⁻^1^ PPFD and above, qN increased significantly under FC_100_ at 70 DAP after planting. Under FC_50_, there was a consistent increase in qN with PPFD across all genotypes. Notably, NM-5 maintained relatively high qN values across both irrigation treatments. Among genotypes, NM-5, NM-6, NM-74, and V-C genotypes showed a generally positive trend in qN, with values of 0.35 to 0.50 at 1000 µmol m⁻^2^ s⁻^1^ PPFD under FC_50_ at 55 and 70 DAP (Table 2). NM-23 exhibited significantly lower qN values than NM 5 and NM 6, particularly under FC_50_. However, qN increased at higher light levels, with a peak of 0.57 at 1000 µmol m⁻^2^ s⁻^1^ PPFD at 70 DAP. Among checks, C-76–16 demonstrated high qN values, particularly under FC_50_, reaching 0.74 at 1000 µmol m⁻^2^ s⁻^1^ PPFD than V-C.

## Discussion

A quadratic polynomial, which explained the gas exchange and chlorophyll fluorescence responses of peanut plants to different PPFD levels, provided an adequate fit for the data, with an R^2^ ranging from 0.88 to 0.99 (Supplementary Table 1). The significant increase in *A* with higher PPFD levels observed across all genotypes and irrigation levels was consistent with the typical response of photosynthetic processes to increased light intensity. Photosynthesis generally increases with light intensity up to a saturation point, after which it may plateau or decline due to limitations in other resources^34,35^. Among the genotypes and irrigation levels, NM-5, NM-70, NM-74, and NM-77 consistently showed significantly higher *A*, particularly at high PPFD levels (Fig. 1). This suggests that these genotypes may have superior photosynthetic efficiency compared to other genotypes. Thus, genotypes with higher photosynthetic rates under deficit irrigations are desirable to breed drought-tolerant genotypes that respond to varying PPFD^36^. Conversely, the decline in the *A* at very high PPFD levels, observed in NM-3, NM-6, and NM-69 genotypes, may be attributed to light saturation or potential photoinhibition effects^37^. However, decline in *A* was further accentuated by reductions in irrigation levels with all the genotypes resulting in higher *A* under FC_100_ than FC_50_. Under FC_50_ with high light intensities, NM-5, NM-70, NM-74, and NM-77 genotypes consistently exhibited higher *A* than other genotypes. Similarly, the current results indicated that higher PPFD levels enhance Tr across the genotypes studied. Genotypes NM-5, NM-74, and NM-77 exhibited significantly higher Tr rates with increased PPFD levels (Table 1). These genotypes exhibit high water-use efficiency that could be linked to physiological adaptations to water deficits^38,39^. Likewise, increased demand for CO_2_ uptake for photosynthesis influences Tr rates under high PPFD levels. These results are consistent with the photosynthetic response of plants to increased light intensity, as higher PPFD generally leads to increased photosynthetic activity and transpiration, driven by greater stomatal conductance and CO₂ uptake^40^. While some genotypes maintained relatively stable Tr rates, others showed marked reductions, indicating varying drought tolerance levels. NM-3 and NM-69 displayed significantly lower Tr rates than other genotypes, suggesting a potentially lower photosynthetic or water use efficiency^40^. Interestingly, V-C showed the highest overall Tr rates, which might reflect a combination of high *A* and water loss under high PPFD conditions. This genotype’s performance underscores its potential for high-light environments and its ability to manage water loss efficiently^41^. On the other hand, C-76–16 consistently had the lowest Tr rates (P < 0.01) among the genotypes, which might indicate a more conservative water use strategy or less efficient photosynthesis under increased light conditions^42^.

Stomatal conductance (*gs*) followed a similar trend, with increases observed at higher PPFD levels, indicating greater stomatal opening to facilitate CO₂ uptake. The results indicate a clear trend of increased stomatal conductance with increasing PPFD across most genotypes. Higher light intensity typically induces significantly higher stomatal conductance to facilitate greater CO₂ uptake, which supports enhanced photosynthetic activity^43–45^. Among genotypes, NM-5 and NM-70 showed increased *gs* with PPFD (Fig. 2), indicating a strong capacity to adapt stomatal opening in response to light availability. This may reflect a high photosynthetic efficiency or a greater ability to balance water loss and CO₂ uptake^46^. C-76–16 exhibited significantly lower *gs* values across all PPFD levels. The significantly low *gs* in NM-69 and NM-74 at high PPFD levels might be indicative of a limitation in their ability to fully utilize increased light for photosynthesis, possibly due to structural or physiological constraints^47,48^. However, under drought stress, stomatal conductance often decreases as a protective mechanism to reduce water loss, which can limit photosynthesis^49^.

The data on electron transport rate (ETR) demonstrates how different genotypes of plants respond to varying light intensities and their efficiency in converting light energy to chemical energy over time. The data suggest that both irrigation strategies influence ETR, but their effects vary significantly across genotypes and environmental conditions. Overall, ETR increases with higher PPFD levels across most genotypes, indicating that the plants can enhance their photosynthetic electron transport as light intensity rises. This finding aligns with previous studies showing that increased light availability can stimulate higher electron transport rates up to a certain threshold^50^. Conversely, NM-5 and NM-74 genotypes displayed enhanced ETR significantly under deficit irrigation, especially at 70 DAP (Fig. 4). Thus, these genotypes may possess mechanisms that optimize photosynthesis when water is limited. For most genotypes, ETR values under full irrigation conditions were significantly higher than those observed under FC_50_, particularly at higher PPFD levels. This decline reflects genotypic sensitivity to water stress, resulting in lower photosynthetic efficiency. Nogues and Baker^51^ indicated that some genotypes are less tolerant to water deficits, affecting their overall photosynthetic capacity. Conversely, genotypes like V-C and C-76–16 show more modest increases in ETR, reflecting potential limitations in their light-harvesting or electron transport capabilities. The decline in ETR at very high PPFD levels, notably observed in NM 69 and NM 70, indicates photoinhibition or saturation of the photosynthetic apparatus. However, significant decrease in ETR at very high PPFD levels at 70 DAP, as compared to 55 DAP was observed, suggests that the plants may experience increased stress or damage to the photosynthetic apparatus over time, affecting their overall efficiency^52^.

The Quantum yield of photosystem II (ΦPSII) reflects the efficiency of converting light energy into chemical energy. It provides insights into the capacity of plants to utilize light energy effectively^50^. Drought stress can further impair quantum yield by causing damage to the photosynthetic apparatus and disrupting the light-harvesting complexes^49,53,54^. NM-5, NM-74, and NM-77 exhibited significantly higher ΦPSII under deficit and fully irrigated conditions than other peanut genotypes (Fig. 3). Thus, these genotypes are more adept at sustaining photosynthetic performance by maintaining higher ETR and ΦPSII during water stress conditions. Sakoda et al.^55^ opined that genotypes maintaining higher *gs* and electron transport rates under varying light conditions might confer advantages in varying environments under changing climatic conditions. Further, FC_100_ led to higher *gs* and ETR among all the genotypes with different light intensities. Concurrently, NM-5, NM-74, and NM-77 genotypes exhibited significantly higher ΦPSII under FC_50_.

Likewise, chlorophyll fluorescence parameters like Fv’/Fm’ denote the maximum quantum efficiency of PSII, which decreased in the current study with higher light intensities, that is between 1000 and 1500 PPFD. This decrease indicates a reduction in the photosynthetic efficiency of PSII, likely due to photoinhibition or stress induced by high light levels. Similar findings were reported by Murchie and Lawson^56^. Among genotypes, NM-5, NM-23, and NM-77 were stable and exhibited significantly higher Fv’/Fm’ values across different PPFD levels (Table 1), suggesting they may possess superior mechanisms for coping with high light intensities^57,58^. Genotypes like NM-3, NM-6, NM-70, and NM-74 exhibit a partial recovery in Fv’/Fm’ at the highest PPFD level (2000 PPFD), suggesting adaptive mechanisms that help mitigate the adverse effects of prolonged high light exposure. Meanwhile, NM-6 and NM-70 show a more significant decrease in Fv’/Fm’ at 70 DAP compared to 55 DAP, indicating potential deterioration in PSII efficiency over time.

Across all genotypes, there is a consistent decrease in qP as PPFD increases. This reduction signifies a decrease in the proportion of open PSII reaction centers and suggests that high light intensities lead to increased photoinhibition or damage to the PSII complexes. Similar trends have been observed in other studies, where high light levels cause a decrease in qP due to saturation and damage to photosynthetic machinery^56^. Genotypes like NM-5, NM-23, NM-74, and NM-77 maintain significantly higher and stable qP values even at high light intensities (Table 2). This variation may reflect differences in light tolerance and adaptation mechanisms among genotypes. Higher qP values at elevated PPFDs in some genotypes may indicate a better ability to sustain open PSII reaction centers under high light stress^37^. For most genotypes, qP decreases from 55 to 70 DAP, particularly at higher PPFD levels. This temporal decline suggests a progressive reduction in photosynthetic efficiency or an increase in photoinhibitory effects over time. This observation is consistent with findings that prolonged high-light exposure can lead to increased photoinhibition and reduced capacity for photosynthesis^59,60^. Likewise, most genotypes exhibit increased qN with higher PPFD, reflecting an enhanced capacity to dissipate excess light energy as heat. Genotypes like NM 69 and V-C showed increasing qN with higher PPFD (Table 2), which indicates efficient energy dissipation mechanisms. This behavior aligns with the role of qN in protecting plants from potential photodamage due to high light intensities^56,61^. Genotypes such as NM-70 and NM-77 exhibited more inconsistent qN patterns, which may reflect either a less effective photoprotective response or potential measurement variations^57^. Genotypes with higher qN values, such as V-C and C-76–16, likely possess more robust mechanisms for dissipating excess light energy. Certain negative or low qN values in some genotypes could point to issues with the measurement or indicate less effective qN. The variability in qN responses across genotypes suggests that selecting optimum qN could be a valuable trait in breeding programs aimed at developing plants with better tolerance to light stress. Adireddy et al.^62^ opined that 50% irrigations exhibited greater qP values in peanut maximizing light use under limited water supply; concurrently, resulting in photoinhibition through reduced *A* and *gs*.

**Fig. 5:**
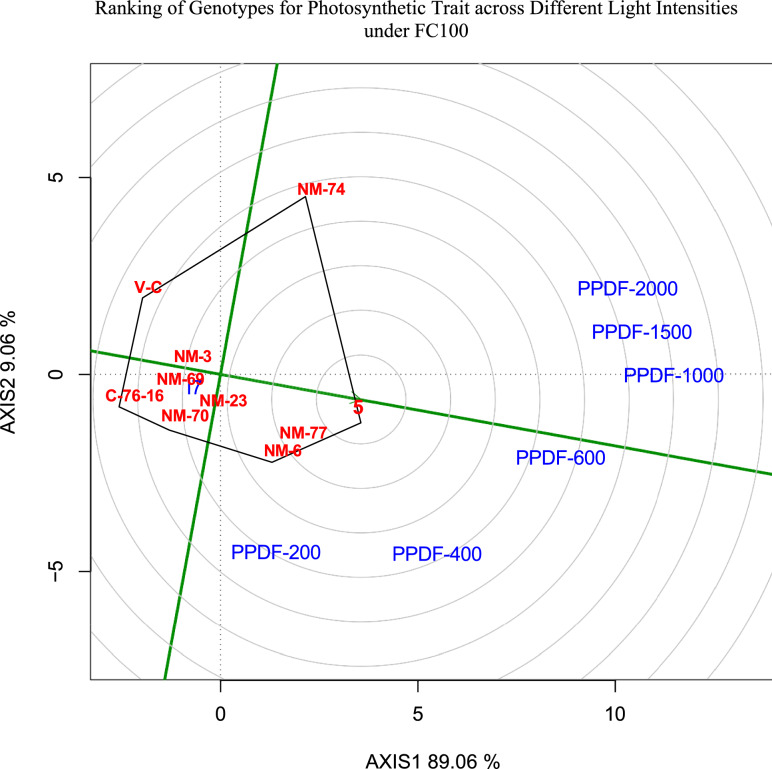
Genotype ranking for photosynthetic rate (*A*) across different PPFD levels at FC_100_.

**Fig. 6:**
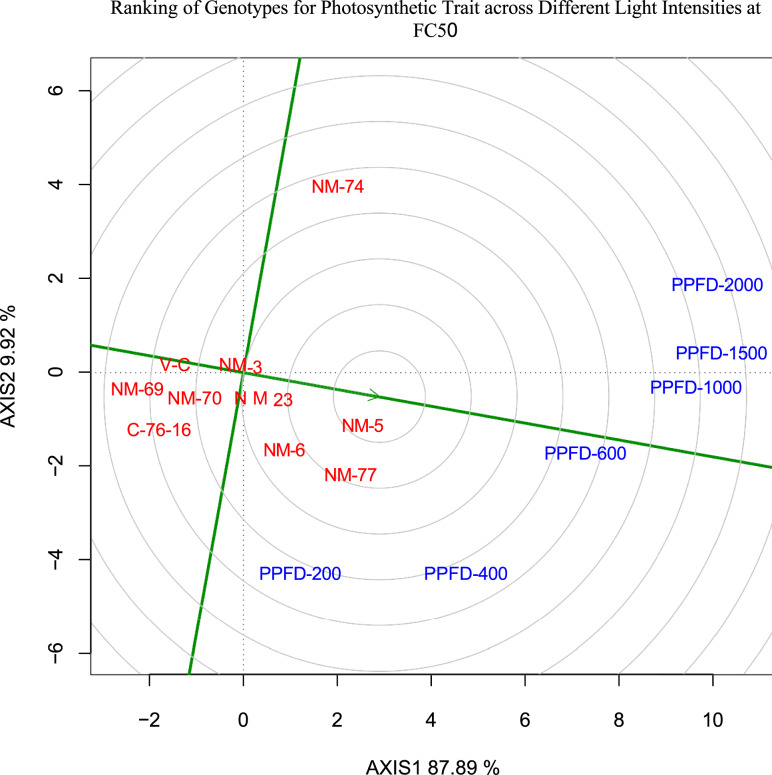
Genotype ranking for photosynthetic rate (*A*) across different PPFD levels at FC_50_.

**Fig. 7:**
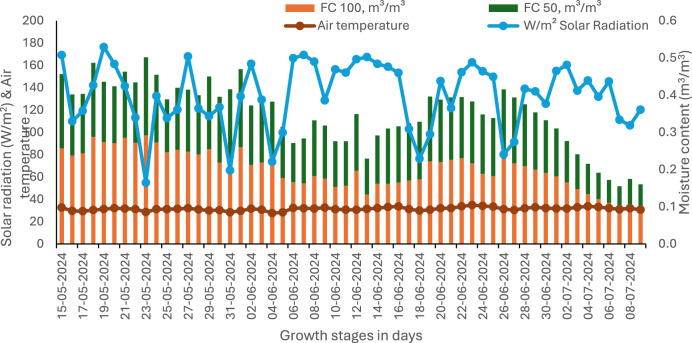
Soil moisture content, solar radiation and air temperature during peanut growth period under two irrigation regimes.

The ANOVA results in Table 3 highlight the effects of genotype, intensity, and their interactions with the irrigation regime (IR) on various physiological traits. Genotype exhibited significant effects (P < 0.01) on multiple traits, including ETR 55, ETR 70, *gs* 55, *gs* 70, PSII 55, PSII 70, qP 55, and qN 55, indicating substantial genetic variability. Intensity had highly significant effects across all traits, demonstrating the strong influence of stress levels. The interaction between genotype and intensity was also significant for several traits, suggesting differential genotypic responses to stress conditions. Additionally, the three-way interaction (Factors × Genotype × Intensity) was significant (P < 0.01) for most parameters, reflecting the complex interplay between these factors. From Figs.5,6, we can conclude that genotype NM-3 was highly stable across different light intensities and has shown good performance, followed by NM-23. Genotypes NM-5, NM-6 and NM-77 were average performance. Genotypes NM-69, NM-70 grouped with two check varieties Valencia-C and C-76–16. The highly significant (p < 0.01) genotype effect for multiple traits indicates a strong genetic basis for these physiological responses, suggesting the potential for genetic improvement through selection. Additionally, the genotype × irrigation interaction was significant for several traits, highlighting the influence of environmental factors on genotype performance. This interaction suggests that genotypes exhibit differential physiological responses under varying conditions, crucial for identifying stress-resilient genotypes suitable for specific environments. The significant genotype effects and interactions indicate that certain genotypes NM-3, NM-5) may possess adaptive traits conferring superior physiological performance under stress conditions to photosynthesis. Among the different light intensities, PPFD-1500, PPFD-1000 can be grouped into Group-1, followed by PPFD-2000 into Group 2, PPFD-600 into Group 3 and PPFD-200 and PPFD-400 into Group 4. With optimum soil moisture contents, solar radiations, and air temperatures, FC_50_ treatment exhibited better *A*, gs, ETR, PSII, qP and qN across all the peanut genotypes (Fig. 7). However, well irrigated pots maintained higher soil moisture content than deficit irrigated pots.

**Table 3:** ANOVA for different photosynthetic attributes.

Source	DF	Tr		*A*		*gs*		*ΦPSII*		ETR		Fv’/Fm’		qP		qN	
		55	70	55	70	55	70	55	70	55	70	55	70	55	70	55	70
Replication	2	10.1	11.8	0.7741	3.7868	0.072	0.1045	0.0016	0.0023	201.9361	538.8	0.0001	0.0069	0.0017	0.0104	0.0016	0.0006
Irrigation level	1	724.4	378.8	510.4465	40.0958	5.0952	2.4748	0.0075	0.0075	1312.351	464.9	0.0053	0.0075	0.0171	0	0.4384	0.239
Error (a)	2	51.8	132.3	91.6921	359.6331	0.3575	0.9921	0.0046	0.0283	792.778	3784.9	0.0004	0.0124	0.009	0.0273	0.0434	0.0967
Genotype	9	239.4**	262*	228.489**	160.449	1.8623**	2.3362**	0.0492**	0.0187**	5392.39**	2287.6**	0.0255**	0.013	0.0741**	0.0301	0.4523**	0.321***
Factors irrigation level × Genotype	9	99.2**	97.3	87.7349	98.7333	0.8349**	1.0046**	0.0176**	0.0134	1047.596	912.5	0.0315**	0.0254**	0.0196**	0.0227	0.2136**	0.1957**
Error (b)	36	8.9	36.1	11.7997	49.6483	0.0815	0.3093	0.0019	0.0079	179.9417	830.6	0.0026	0.0085	0.0032	0.0146	0.0146	0.0564
PPFD	6	157.9**	196.3**	6723.7244**	6713.2461**	0.5955**	0.766**	3.4345**	3.4625**	122,191.3**	118,725.0**	3.9111**	3.9924**	6.7454**	6.6994**	1.9288**	1.7849**
Factors: PPFD	6	7.2**	10.9**	68.4461**	34.7759**	0.0375**	0.0627**	0.0017**	0.0021**	342.5698**	40.2	0.0026**	0.0029**	0.0009	0	0.0391**	0.0429**
Genotype × PPFD	54	6.4**	5**	26.8133**	18.7966**	0.0391**	0.0299**	0.0013**	0.0013**	591.2149**	332.8**	0.0016**	0.0012**	0.0057**	0.0023*	0.0312**	0.0231**
Factors: Genotype × PPFD	54	1.4**	1.5**	9.7738**	6.7305*	0.0082**	0.01**	0.0012**	0.0012**	134.2879**	105.2	0.0017**	0.0016**	0.0024**	0.0027**	0.0204**	0.0235**
Error (c)	240	0.2	0.6	1.1757	4.7219	0.0009	0.0032	0.0006	0.0006	23.2991	103.5	0.0001	0.0005	0.0004	0.0015	0.0017	0.0064

Tr, transpiration rate; A, photosynthetic rate; *gs*, stomatal conductivity; *ΦPSII,* quantum yield of PSII; ETR, electron transport rate; qP, photochemical quenching; qN, non-photochemical quenching; 55 and 70 represents days after sowing; * denotes statistical significance at p < 0.05; ** denotes statistical significance at p < 0.01.

## Conclusion

A key focus in assessing peanut genotype productivity under drought stress is investigating its photosynthetic efficiencies in naturally varying light intensities encountered in the field. Among ten peanut genotypes assessed in this study, NM-5, NM-74, and NM-77 maintained higher *A*, *gs*, ΦPSII, and ETR under higher light conditions possible during the day; as such, they are possibly more suited than others for growing in drought-stressed environments. Variabilities among genotypes in responding to different light intensities indicate that some genotypes are more adapted to high light than others, which could be leveraged in breeding programs for improved performance under varying light intensities. More investigation into the physiological mechanisms behind these variabilities may be necessary to improve the peanut breeder’s comprehension of plant-environment interactions for developing novel genotypes for superior performance under water-limited environments.

## Materials and methods

### Breeding lines and study setup

The environment-controlled greenhouse study was carried out from March to July 2024 at the Crop Production Systems Research Unit, United States Department of Agriculture, Stoneville, MS, USA. Plants were grown in an environmentally controlled greenhouse with day/night temperatures of 35/20⁰ C with 60% relative humidity. The minimum daily photosynthetic photon flux density was maintained at 900 ± 20 μmol/m/s using sodium vapor lamps. Ten different peanut genotypes used in this study represent the four market types: Spanish, Virginia, Runner, and Valencia. Investigated were eight newly developed genotypes in the New Mexico (NM) series [NM-3, NM-5, NM-6, NM-23, NM-69, NM-70, NM-74, and NM-77], and they were compared with the check varieties Valencia-C (V-C) and C-76–16. These genotypes and varieties were obtained from Department of Plant and Environmental Sciences, New Mexico State University, Clovis—New Mexico- 88,101, USA. The pots were arranged in a split-pots design with two soil water levels: 1) 100% field capacity (FC_100_) and 2) 50% field capacity (FC_50_) as main plots. The genotypes were randomized in sub-pots with four replications. Soil from farm fields at the location was used in the experiment. The soil type was a Bosket, very fine sandy loam with 8.0 pH, 0.60% organic carbon, 0.5 g kg^−1^ total N, 96 kg ha^−1^ available P, and 265 kg ha^−1^ available K. About 14 kg of soil has been filled in near-cylindrical pots with 28 cm diameter and 30 cm depth. Two seeds of each genotype were planted in each pot on March 18, 2024. Fifteen days after emergence, seedlings in each pot were thinned to one seedling per pot. The volumetric soil water content determined at the field capacity was 40.8%. Watering at FC_100_ was provided every two days, and irrigation at FC_50_ was provided every four days. Soil moisture content was monitored in each pot using a soil water content sensor (TEROS 10, Meter, WA, USA) at 10 cm below the surface. Average soil water contents measured 24 h after irrigation was used as field capacity for irrigation. The soil moisture content during the crop growth period is depicted in Fig. 7. Thirty days after planting (DAP), Miracle Grow (24:8:16% N:P: K) NPK fertilizer was evenly applied to each pot as minor leaf yellowing was observed. The air temperature and solar radiation were measured using a barometer-enabled automatic weather station from sowing to harvesting of peanut genotypes, and data is presented in Fig. 7.

### Leaf gas exchange parameters

A LI 6800-01A photosynthetic system integrated fluorometer (LI-COR, NE, USA) was used to measure gas exchange parameters: net photosynthetic rate (*A*), transpiration (Tr), and stomatal conductance (*gs*). The LI 6800 photosynthetic system integrated fluorometer (LI-COR, Inc., USA) uses a ‘dark pulse’ routine to determine Fo and Fm in a light-adapted leaf (LI-COR, Inc. 2019). In this procedure, on a light-adapted leaf, a brief pulse of far-red light was used to excite PSI, and electrons were forced to drain from PSII, mimicking dark adaptation before measurements were taken. Measurements were made on the third completely opened quadrifoliate leaf between 10:00 a.m. and 2:00 p.m. in response to Photosynthetic Photon Flux Densities (PPFD) 0, 200, 400, 600, 1000, 1500, and 2000 μ mol m^−2^ s^−1^. Constant maximum and minimum setpoints for steady-state stable photosynthetic responses was 70 s, and the response averaging time was 4 s. For each measurement, a single leaf from a single plant in each treatment under three replications was used. For leaf gas exchange measurements, air CO_2_ concentration was maintained at 420 ppm, relative humidity at 50%, and airflow rate at 600 μmol s^−1^. Gas exchange parameters like *A*, *gs*, Tr, chlorophyll fluorescence parameters like maximum fluorescence, and photochemical and non-photochemical quenching were quantified in response to light. The LI 6800 photosynthetic system integrated fluorometer (LI-COR, Inc., USA) uses a ‘dark pulse’ routine to determine Fo and Fm in a light-adapted leaf (LI-COR, Inc. 2019). In this procedure, on a light-adapted leaf, a brief pulse of far-red light was used to excite PSI, and electrons were forced to drain from PSII, mimicking dark adaptation before measurements were taken. The airflow rate into the LI 6800 chamber was 600 μ mol s^−1^, RH was 50%, and the air temperature was set to the measured leaf temperature. Measurements were conducted two days after irrigations on 55 and 70 DAP, as these days corresponds to maximum flowering and pod development stages in peanut^63^. The data on Fo, Fm, Fv/Fm, vapor pressure deficit in leaves and water use efficiency of different genotypes have been given in Supplementary Table 2a,b.

### Statistical analysis

A second-order polynomial best fitted the light response curves, gas exchange, and chlorophyll fluorescence parameters plotted against light applied for the 10 genotypes with two irrigation levels. The fitted regression lines were assessed using analysis of covariance (ANCOVA)^64^. The R^2^ values with polynomial equations for the gas exchange and chlorophyll fluorescence were given in Supplementary Table 1. The data analysis was conducted using STAR software, GPL-2.0 (https://github.com/angell1117/STAR-genome-browser) for a split-plot design, where irrigation levels were considered as the main plot factor, while genotypes, light intensity, and replications were treated as sub-factors or subplots. The dataset was first structured appropriately and imported into STAR, where the split-plot design was defined, specifying irrigation as the main factor and genotypes and light intensity as subplot factors. Analysis of Variance (ANOVA) was performed to assess the significance of these factors and their interactions. Principal Component Analysis (PCA) was conducted in R to evaluate the variation in photosynthesis-related traits among genotypes under different conditions. The analysis was performed using the FactoMineR and factoextra packages. Additionally, GGE biplot analysis was conducted in R to evaluate genotype-by-environment interactions, considering photosynthesis as a critical trait using GGEBiplotGUI package. GGE model was created, and the “Which-won-where” biplot was generated to identify superior genotypes under specific irrigation conditions. The ranking of genotypes was also analyzed to determine their stability and adaptability in different environments.

## Supplementary Information

Supplementary Information.

## Data Availability

Data is provided within the manuscript.
