# Supplementary material for: Photosynthetic responses to light levels in drought-tolerant novel peanut (Arachis hypogaea L) genotypes
Source: Sci Rep. 2025 Jul 11;15:25032. doi: 10.1038/s41598-025-10978-z (PMC12254281; doi:10.1038/s41598-025-10978-z)
Supplement: Supplementary file 1 — Supplementary Information. [file 41598_2025_10978_MOESM1_ESM.docx]

Supplementary table 1: Polynomial equations for photosynthetic process of different genotypes under different irrigation levels.

| Genotype | Full irrigation | Deficit irrigation |
| --- | --- | --- |
| *Photosynthetic rate* | | |
| NM3 | FI 55 = -1.1572x2 + 12.949x - 12.244 R² = 0.984 | DI 55= -1.2147x2 + 14.067x - 13.247 R² = 0.984 |
|  | FI 70= -1.1646x2 + 12.946x - 13.463 R² = 0.995 | DI-70 = -1.2891x2 + 15.345x - 14.378 R² = 0.98 |
| NM 5 | FI 55= -1.3005x^2^ + 16.862x - 17.721 R² = 0.9965 | DI 55 = -1.4123x^2^ + 16.416x - 15.271 R² = 0.9841 |
|  | FI 70 = -1.3594x^2^ + 16.172x - 14.9 R² = 0.9786 | DI 70 = -1.3005x^2^ + 16.862x - 17.721 R² = 0.9965 |
| NM 6 | FI 55 = -1.437x^2^ + 16.498x - 15.219 R² = 0.9865 | DI 55 = -1.1489x^2^ + 13.455x - 11.463 R² = 0.968 |
|  | FI 70 = -1.3248x^2^ + 15.335x - 13.943 R² = 0.9799 | DI 70= -1.0569x^2^ + 12.554x - 10.495 R² = 0.9543 |
| NM 23 | FI 55 = -1.2899x^2^ + 15.086x - 14.126 R² = 0.9852 | DI 55 = -1.0653x^2^ + 12.55x - 10.942 R² = 0.9613 |
|  | FI 70 = -1.194x^2^ + 14.198x - 13.248 R² = 0.981 | DI 70 = -0.9904x^2^ + 11.705x - 9.8803 R² = 0.9478 |
| NM 69 | FI 55 = -1.2662x^2^ + 14.608x - 13.597 R² = 0.9799 | I 55 = -0.9154x^2^ + 10.436x - 8.5524 R² = 0.9229 |
|  | FI 70 = -1.2613x^2^ + 14.048x - 12.892 R² = 0.976 | DI 70 = -0.7985x^2^ + 9.0453x - 6.8708 R² = 0.8786 |
| NM 70 | FI 55 = -1.1023x^2^ + 14.097x - 13.678 R² = 0.9912 | DI 55 = -1.39x^2^ + 13.227x - 10.77 R² = 0.882 |
|  | FI 70= -0.8882x^2^ + 11.789x - 11.743 R² = 0.991 | DI 70= -1.3349x^2^ + 14.249x - 11.936 R² = 0.9266 |
| NM 74 | FI 55 = -1.1712x^2^ + 15.764x - 16.784 R² = 0.9994 | DI 55 = -0.4959x^2^ + 9.4387x - 10.128 R² = 0.9984 |
|  | FI 70 = -0.9824x^2^ + 13.949x - 15.761 R² = 0.9984 | DI 70 = -0.7521x^2^ + 11.296x - 11.156 R² = 0.9897 |
| NM 77 | FI 55 = -1.1738x^2^ + 14.528x - 13.317 R² = 0.9742 | DI 55 = -1.3591x^2^ + 15.531x - 14.223 R² = 0.9825 |
|  | FI 70 = -1.2619x^2^ + 15.525x - 14.734 | DI 70 = -1.4754x^2^ + 16.18x - 14.911 R² = 0.9837 |
| V-C | FI 55 = -1.0393x^2^ + 12.228x - 13.211 R² = 0.9961 | DI 55 = -1.1752x^2^ + 13.235x - 12.351 R² = 0.9841 |
|  | FI 70 = -1.1349x^2^ + 13.01x - 13.338 R² = 0.9928 | DI 70 = -1.1843x^2^ + 13.167x - 11.586 R² = 0.9673 |
| C-76-16 | FI 55 = -1.1515x^2^ + 13.005x - 11.334 R² = 0.9661 | DI 55 = -0.8578x^2^ + 9.4921x - 6.3162 R² = 0.8491 |
|  | FI 70 = -1.1955x^2^ + 14.002x - 12.87 R² = 0.9771 | DI 70 = -0.8995x^2^ + 10.735x - 8.3976 R² = 0.9352 |
| *Stomatal conductance* | | |
| NM3 | FI 55 = -0.0044x^2^ + 0.102x + 0.158 R² = 0.9941 | DI 55 = -0.0082x^2^ + 0.1549x + 0.1249 R² = 0.9869 |
|  | FI 70 = -0.0056x^2^ + 0.1226x + 0.0837 R² = 0.9883 | DI 70 = -0.0136x^2^ + 0.2012x + 0.16 R² = 0.9982 |
| NM 5 | FI 55 = -0.006x^2^ + 0.1643x + 0.4132 R² = 0.9601 | DI 55 = -0.0212x^2^ + 0.2355x + 0.6802 R² = 0.9838 |
|  | FI 70 = -0.0176x^2^ + 0.2762x + 0.172 R² = 0.9947 | DI 70 = -0.0179x^2^ + 0.1825x + 1.0577 R² = 0.9475 |
| NM 6 | FI 55 = -0.0103x^2^ + 0.1158x + 0.4323 R² = 0.998 | DI 55 = -0.0069x^2^ + 0.0965x + 0.4712 R² = 0.989 |
|  | FI 70 = -0.0087x^2^ + 0.1097x + 0.3871 R² = 0.996 | DI 70 = -0.0018x^2^ + 0.0538x + 0.3945 R² = 0.986 |
| NM 23 | FI 55 = -0.0057x^2^ + 0.0875x + 0.4868 R² = 0.999 | DI 55 = 0.005x^2^ - 0.0078x + 0.3477 R² = 0.968 |
|  | FI 70 = -0.0061x^2^ + 0.0993x + 0.4974 R² = 0.998 | DI 70 = 0.0085x^2^ - 0.0396x + 0.3013 R² = 0.942 |
| NM 69 | FI 55 = -0.0037x^2^ + 0.0488x + 0.472 R² = 0.980 | DI 55 = 0.0016x^2^ - 0.0057x + 0.2649 R² = 0.97 |
|  | FI 70 = -0.0042x^2^ + 0.0535x + 0.3609 R² = 0.989 | DI 70 = 0.0033x^2^ - 0.0239x + 0.1959 R² = 0.877 |
| NM 70 | FI 55 = -0.0092x^2^ + 0.1364x + 0.7391 R² = 0.998 | DI 55 = -0.0024x^2^ + 0.0068x + 0.4343 R² = 0.808 |
|  | FI 70 = -0.006x^2^ + 0.1297x + 0.4764 R² = 0.996 | DI 70 = -0.0051x^2^ + 0.0609x + 0.5521 R² = 0.971 |
| NM 74 | FI 55 = -0.007x^2^ + 0.1701x + 0.1738 R² = 0.993 | DI 55 = 0.0046x^2^ + 0.0506x + 0.0891 R² = 0.999 |
|  | FI 70 = -0.0017x^2^ + 0.1444x + 0.0751 R² = 0.981 | DI 70 = -0.002x^2^ + 0.0837x + 0.2297 R² = 0.993 |
| NM 77 | FI 55 = -0.0086x^2^ + 0.1116x + 0.5572 R² = 0.993 | DI 55 = -0.0065x^2^ + 0.0859x + 0.2973 R² = 0.986 |
|  | FI 70 = -0.013x^2^ + 0.1695x + 0.5433 R² = 0.995 | DI 70 = -0.006x^2^ + 0.0978x + 0.2757 R² = 0.9843 |
| V-C | FI 55 = -0.0038x^2^ + 0.049x + 0.9198 R² = 0.987 | DI 55 = -0.0021x^2^ + 0.026x + 0.5455 R² = 0.990 |
|  | FI 70 = -0.0037x^2^ + 0.0487x + 0.7724 R² = 0.988 | DI 70 = -0.0009x^2^ + 0.033x + 0.4286 R² = 0.981 |
| C-76-16 | FI 55 = -0.0018x^2^ + 0.039x + 0.3074 R² = 0.986 | DI 55 = 0.0045x^2^ - 0.0453x + 0.3106 R² = 0.713 |
|  | FI 70 = -0.003x^2^ + 0.0597x + 0.4013 R² = 0.993 | DI 70 = 0.0043x^2^ - 0.0296x + 0.2576 R² = 0.655 |
| *ФPS2 Quantum yield* | | |
| NM3 | FI 55 = 0.0074x^2^ - 0.1591x + 0.7811 R² = 0.990 | DI 55 = 0.0067x^2^ - 0.1611x + 0.8323 R² = 0.987 |
|  | FI 70 = 0.0044x^2^ - 0.1278x + 0.6936 R² = 0.993 | DI 70 = 0.0061x^2^ - 0.1575x + 0.8513 R² = 0.982 |
| NM 5 | FI 55 = -0.001x^2^ - 0.0968x + 0.7959 R² = 0.986 | DI 55 = 0.0061x^2^ - 0.1544x + 0.8519 R² = 0.989 |
|  | FI 70 = 0.0029x^2^ - 0.1286x + 0.8168 R² = 0.986 | DI 70 = 0.0077x^2^ - 0.1681x + 0.8718 R² = 0.988 |
| NM 6 | FI 55 = 0.0013x^2^ - 0.124x + 0.8208 R² = 0.985 | DI 55 = 0.0106x^2^ - 0.1885x + 0.8671 R² = 0.993 |
|  | FI 70 = 0.0045x^2^ - 0.1484x + 0.8411 R² = 0.989 | DI 70 = 0.0108x^2^ - 0.1886x + 0.8704 R² = 0.991 |
| NM 23 | FI 55 = 0.0054x^2^ - 0.1543x + 0.8459 R² = 0.988 | DI 55 = 0.0072x^2^ - 0.1703x + 0.8716 R² = 0.988 |
|  | FI 70 = 0.0082x^2^ - 0.1733x + 0.8566 R² = 0.991 | DI 70 = 0.0073x^2^ - 0.1706x + 0.8704 R² = 0.987 |
| NM 69 | FI 55 = 0.0037x^2^ - 0.1439x + 0.8466 R² = 0.988 | DI 55 = 0.0015x^2^ - 0.1332x + 0.8311 R² = 0.994 |
|  | FI 70 = 0.0047x^2^ - 0.1536x + 0.8508 R² = 0.992 | DI 70 = 0.0007x^2^ - 0.1296x + 0.826 R² = 0.995 |
| NM 70 | FI 55 = 0.007x^2^ - 0.159x + 0.8271 R² = 0.994 | DI 55 = -0.0014x^2^ - 0.1169x + 0.8165 R² = 0.988 |
|  | FI 70 = 0.0106x^2^ - 0.1805x + 0.8106 R² = 0.997 | DI 70 = 0.0007x^2^ - 0.1267x + 0.8355 R² = 0.985 |
| NM 74 | FI 55 = -0.0002x^2^ - 0.1062x + 0.8099 R² = 0.985 | DI 55 = 0.0011x^2^ - 0.1155x + 0.8143 R² = 0.983 |
|  | FI 70 = 0.0023x^2^ - 0.1216x + 0.8004 R² = 0.989 | DI 70 = 0.0043x^2^ - 0.14x + 0.8386 R² = 0.985 |
| NM 77 | FI 55 = 0.0053x^2^ - 0.1474x + 0.8298 R² = 0.985 | DI 55 = 0.0041x^2^ - 0.1448x + 0.8428 R² = 0.991 |
|  | FI 70 = 0.0033x^2^ - 0.1344x + 0.8253 R² = 0.984 | DI 70 = 0.0054x^2^ - 0.1559x + 0.8568 R² = 0.992 |
| V-C | FI 55 = 0.0086x^2^ - 0.144x + 0.6607 R² = 0.993 | DI 55 = 0.0078x^2^ - 0.1609x + 0.8019 R² = 0.990 |
|  | FI 70 = 0.0086x^2^ - 0.1555x + 0.7269 R² = 0.991 | DI 70 = 0.0101x^2^ - 0.1854x + 0.8554 R² = 0.992 |
| C-76-16 | FI 55 = 0.0065x^2^ - 0.164x + 0.8401 R² = 0.993 | DI 55 = 0.0182x^2^ - 0.2291x + 0.7766 R² = 0.999 |
|  | FI 70 = 0.0053x^2^ - 0.1536x + 0.8366 R² = 0.992 | DI 70 = 0.0111x^2^ - 0.1803x + 0.7825 R² = 0.999 |
| *Electron transport rate* | | |
| NM3 | FI 55 = -4.9759x^2^ + 41.323x + 18.403 R² = 0.971 | DI 55 = -5.2019x^2^ + 44.847x + 19.76 R² = 0.957 |
|  | FI 70 = -5.5393x^2^ + 45.097x + 8.43 R² = 0.993 | DI 70 = -4.8097x^2^ + 44.95x + 21.775 R² = 0.95 |
| NM 5 | FI 55 = -5.4799x^2^ + 59.686x + 4.1178 R² = 0.998 | DI 55 = -2.7093x^2^ + 30.806x + 49.404 R² = 0.996 |
|  | FI 70 = -5.1086x^2^ + 51.595x + 12.801 R² = 0.992 | DI 70 = -4.2403x^2^ + 42.855x + 24.035 R² = 0.967 |
| NM 6 | FI 55 = -6.7522x^2^ + 59.031x + 6.8555 R² = 0.994 | DI 55 = -4.2867x^2^ + 38.992x + 25.715 R² = 0.968 |
|  | FI 70 = -6.1766x^2^ + 53.04x + 12.787 R² = 0.989 | DI 70 = -3.606x^2^ + 36.092x + 28.945 R² = 0.956 |
| NM 23 | FI 55 = -5.8152x^2^ + 50.169x + 15.931 R² = 0.979 | DI 55 = -3.713x^2^ + 31.754x + 50.066 R² = 0.983 |
|  | FI 70 = -4.9201x^2^ + 43.382x + 22.06 R² = 0.966 | DI 70 = -5.3933x^2^ + 46.092x + 21.443 R² = 0.949 |
| NM 69 | FI 55 = -6.3511x^2^ + 54.949x + 11.824 R² = 0.990 | DI 55 = -8.8849x^2^ + 67.488x - 1.4271 R² = 0.980 |
|  | FI 70 = -7.1083x^2^ + 56.546x + 9.9666 R² = 0.992 | DI 70 = -9.7295x^2^ + 71.667x - 5.844 R² = 0.965 |
| NM 70 | FI 55 = -4.9262x^2^ + 45.763x + 17.026 R² = 0.992 | DI 55 = -9.9648x^2^ + 72.584x - 5.1929 R² = 0.999 |
|  | FI 70 = -4.0438x^2^ + 36.679x + 22.796 R² = 0.987 | DI 70 = -7.8656x^2^ + 63.309x + 4.5255 R² = 0.994 |
| NM 74 | FI 55 = -5.7338x^2^ + 59.141x + 5.7283 R² = 0.998 | DI 55 = -5.2009x^2^ + 54.765x + 10.125 R² = 0.994 |
|  | FI 70 = -3.3973x^2^ + 38.038x + 39.156 R² = 0.986 | DI 70 = -4.6085x^2^ + 48.365x + 17.284 R² = 0.986 |
| NM 77 | FI 55 = -4.7461x^2^ + 46.595x + 17.931 R² = 0.979 | DI 55 = -6.2381x^2^ + 54.86x + 10.885 R² = 0.993 |
|  | FI 70 = -5.3309x^2^ + 51.37x + 13.802 R² = 0.988 | DI 70 = -6.262x^2^ + 53.473x + 12.865 R² = 0.990 |
| V-C | FI 55 = -2.2758x^2^ + 25.66x + 23.291 R² = 0.965 | DI 55 = -4.1824x^2^ + 39.34x + 21.77 R² = 0.969 |
|  | FI 70 = -3.2096x^2^ + 31.263x + 23.334 R² = 0.956 | DI 70 = -4.4848x^2^ + 39.075x + 25.266 R² = 0.956 |
| C-76-16 | FI 55 = -6.6232x^2^ + 51.66x + 13.033 R² = 0.989 | DI 55 = -4.8015x^2^ + 38.099x + 12.813 R² = 0.959 |
|  | FI 70 = -6.1912x^2^ + 51.911x + 13.012 R² = 0.992 | DI 70 = -5.1465x^2^ + 46.754x + 8.7425 R² = 0.995 |

Supplementary table 2a: Photosynthetic process, water use efficiency and vapor pressure deficit among peanut genotypes at 55 days after sowing under full and deficit irrigated condition

| Genotype | PPFD | A  (µ mol m^-2^ s^-1^) | ΦPSII | WUE  (µmol CO_2_/m  mol H_2_O) | VPD leaf (kpa) | Fo | Fm | Fs | Fm' | Fo' |
| --- | --- | --- | --- | --- | --- | --- | --- | --- | --- | --- |
| *Full irrigation* | | | | | | | | | | |
| NM-3 | 2000 | 22.48 | 0.11 | 2.18 | 1.70 | 296.44 | 820.61 | 639.5 | 717.6 | 308.3 |
|  | 1500 | 23.40 | 0.16 | 2.47 | 1.61 | 296.44 | 820.61 | 526.8 | 625.0 | 289.8 |
|  | 1000 | 22.43 | 0.24 | 2.69 | 1.54 | 296.44 | 820.61 | 442.4 | 582.1 | 277.9 |
|  | 600 | 20.29 | 0.39 | 2.85 | 1.51 | 296.44 | 820.61 | 388.7 | 639.7 | 288.9 |
|  | 400 | 17.56 | 0.51 | 2.92 | 1.50 | 296.44 | 820.61 | 363.5 | 745.2 | 310.6 |
|  | 200 | 10.41 | 0.62 | 2.16 | 1.49 | 296.44 | 820.61 | 320.6 | 846.7 | 328.1 |
|  | 0 | -1.69 | - | -0.44 | 1.47 | 316.10 | 943.59 | 0.0 | 0.0 | - |
| NM-5 | 2000 | 37.29 | 0.20 | 2.26 | 1.52 | 198.99 | 886.35 | 591.3 | 727.5 | 210.9 |
|  | 1500 | 35.96 | 0.26 | 2.39 | 1.46 | 198.99 | 886.35 | 503.4 | 676.1 | 207.0 |
|  | 1000 | 33.59 | 0.37 | 2.43 | 1.41 | 198.99 | 886.35 | 421.3 | 671.6 | 206.6 |
|  | 600 | 28.17 | 0.52 | 2.21 | 1.40 | 198.99 | 886.35 | 357.3 | 755.5 | 213.0 |
|  | 400 | 22.41 | 0.62 | 2.05 | 1.39 | 198.99 | 886.35 | 321.9 | 853.3 | 219.8 |
|  | 200 | 11.59 | 0.68 | 1.40 | 1.42 | 198.99 | 886.35 | 296.7 | 935.6 | 225.4 |
|  | 0 | -3.01 | - | -0.49 | 1.43 | 213.21 | 975.22 | 0.0 | 0.0 | - |
| NM-6 | 2000 | 30.97 | 0.14 | 2.72 | 1.69 | 249.55 | 928.67 | 700.7 | 816.0 | 261.5 |
|  | 1500 | 31.28 | 0.21 | 2.86 | 1.60 | 249.55 | 928.67 | 552.2 | 700.4 | 247.3 |
|  | 1000 | 29.88 | 0.32 | 2.88 | 1.52 | 249.55 | 928.67 | 450.2 | 658.6 | 240.7 |
|  | 600 | 27.07 | 0.49 | 2.79 | 1.47 | 249.55 | 928.67 | 372.8 | 731.5 | 248.8 |
|  | 400 | 23.22 | 0.60 | 2.57 | 1.44 | 249.55 | 928.67 | 339.3 | 859.1 | 263.3 |
|  | 200 | 13.42 | 0.68 | 1.65 | 1.42 | 249.55 | 928.67 | 312.5 | 970.1 | 277.5 |
|  | 0 | -1.60 | - | -0.22 | 1.42 | 252.81 | 1025.59 | 0.0 | 0.0 | - |
| NM-23 | 2000 | 29.40 | 0.13 | 2.52 | 1.56 | 220.33 | 1065.42 | 812.6 | 935.6 | 232.3 |
|  | 1500 | 29.12 | 0.19 | 2.65 | 1.49 | 220.33 | 1065.42 | 679.7 | 835.0 | 225.5 |
|  | 1000 | 27.65 | 0.29 | 2.68 | 1.43 | 220.33 | 1065.42 | 533.9 | 751.6 | 219.0 |
|  | 600 | 24.95 | 0.46 | 2.59 | 1.39 | 220.33 | 1065.42 | 412.4 | 763.0 | 220.0 |
|  | 400 | 21.20 | 0.58 | 2.34 | 1.39 | 220.33 | 1065.42 | 344.9 | 822.9 | 224.7 |
|  | 200 | 12.41 | 0.68 | 1.50 | 1.38 | 220.33 | 1065.42 | 292.5 | 912.2 | 230.8 |
|  | 0 | -1.80 | - | -0.24 | 1.38 | 238.41 | 1098.65 | 0.0 | 0.0 | - |
| NM-69 | 2000 | 27.76 | 0.14 | 2.87 | 1.71 | 206.33 | 782.42 | 614.0 | 713.4 | 218.4 |
|  | 1500 | 27.79 | 0.20 | 2.95 | 1.62 | 206.33 | 782.42 | 486.8 | 609.7 | 207.5 |
|  | 1000 | 26.15 | 0.31 | 2.97 | 1.54 | 206.33 | 782.42 | 405.4 | 584.6 | 204.8 |
|  | 600 | 23.77 | 0.47 | 2.87 | 1.48 | 206.33 | 782.42 | 343.0 | 651.3 | 212.6 |
|  | 400 | 20.73 | 0.60 | 2.61 | 1.48 | 206.33 | 782.42 | 317.4 | 788.0 | 225.5 |
|  | 200 | 12.27 | 0.69 | 1.62 | 1.46 | 206.33 | 782.42 | 291.1 | 931.8 | 236.0 |
|  | 0 | -1.89 | - | -0.27 | 1.43 | 224.01 | 906.19 | 0.0 | 0.0 | - |
| NM-70 | 2000 | 31.29 | 0.14 | 1.97 | 1.53 | 224.11 | 1022.82 | 777.2 | 900.0 | 236.1 |
|  | 1500 | 31.62 | 0.19 | 2.14 | 1.47 | 224.11 | 1022.82 | 649.7 | 805.1 | 229.0 |
|  | 1000 | 28.24 | 0.29 | 2.05 | 1.41 | 224.11 | 1022.82 | 518.3 | 728.1 | 222.3 |
|  | 600 | 23.77 | 0.43 | 1.85 | 1.38 | 224.11 | 1022.82 | 388.2 | 688.1 | 218.1 |
|  | 400 | 20.09 | 0.55 | 1.71 | 1.37 | 224.11 | 1022.82 | 320.7 | 732.9 | 221.8 |
|  | 200 | 11.42 | 0.66 | 1.05 | 1.37 | 224.11 | 1022.82 | 294.0 | 886.1 | 234.5 |
|  | 0 | -1.79 | - | -0.18 | 1.37 | 248.30 | 1085.91 | 0.0 | 0.0 | - |
| NM-74 | 2000 | 36.55 | 0.19 | 2.41 | 1.77 | 215.88 | 813.54 | 613.5 | 757.4 | 227.9 |
|  | 1500 | 35.09 | 0.25 | 2.55 | 1.67 | 215.88 | 813.54 | 539.0 | 719.7 | 224.3 |
|  | 1000 | 32.63 | 0.36 | 2.67 | 1.60 | 215.88 | 813.54 | 450.3 | 712.7 | 224.0 |
|  | 600 | 27.51 | 0.52 | 2.64 | 1.57 | 215.88 | 813.54 | 363.0 | 774.3 | 230.4 |
|  | 400 | 20.51 | 0.61 | 2.47 | 1.59 | 215.88 | 813.54 | 318.5 | 840.5 | 235.3 |
|  | 200 | 9.97 | 0.69 | 1.50 | 1.58 | 215.88 | 813.54 | 295.7 | 956.4 | 246.3 |
|  | 0 | -2.34 | - | -0.44 | 1.55 | 223.31 | 923.79 | 0.0 | 0.0 | - |
| NM-77 | 2000 | 32.12 | 0.15 | 2.45 | 1.59 | 220.22 | 775.62 | 573.4 | 677.6 | 232.2 |
|  | 1500 | 31.29 | 0.21 | 2.57 | 1.48 | 220.22 | 775.62 | 497.2 | 631.0 | 226.4 |
|  | 1000 | 27.19 | 0.29 | 2.36 | 1.41 | 220.22 | 775.62 | 449.3 | 638.0 | 228.1 |
|  | 600 | 25.44 | 0.46 | 2.37 | 1.36 | 220.22 | 775.62 | 371.5 | 694.0 | 235.5 |
|  | 400 | 22.30 | 0.58 | 2.23 | 1.34 | 220.22 | 775.62 | 341.3 | 818.9 | 248.8 |
|  | 200 | 12.70 | 0.67 | 1.41 | 1.34 | 220.22 | 775.62 | 307.0 | 925.6 | 257.9 |
|  | 0 | -1.80 | - | -0.22 | 1.34 | 225.81 | 891.17 | 0.0 | 0.0 | - |
| V-C | 2000 | 21.32 | 0.12 | 1.51 | 1.60 | 212.11 | 668.58 | 507.8 | 575.0 | 224.1 |
|  | 1500 | 23.37 | 0.14 | 1.74 | 1.50 | 212.11 | 668.58 | 502.0 | 587.1 | 228.2 |
|  | 1000 | 21.55 | 0.21 | 1.69 | 1.43 | 212.11 | 668.58 | 456.9 | 577.3 | 227.7 |
|  | 600 | 18.29 | 0.32 | 1.49 | 1.39 | 212.11 | 668.58 | 418.1 | 618.5 | 234.2 |
|  | 400 | 14.59 | 0.42 | 1.21 | 1.38 | 212.11 | 668.58 | 397.3 | 686.0 | 242.9 |
|  | 200 | 7.75 | 0.52 | 0.67 | 1.35 | 212.11 | 668.58 | 373.7 | 780.1 | 253.7 |
|  | 0 | -2.47 | - | -0.22 | 1.33 | 222.21 | 735.22 | 0.0 | 0.0 | - |
| C-76-16 | 2000 | 24.67 | 0.10 | 3.22 | 1.66 | 212.77 | 796.35 | 660.5 | 737.8 | 224.8 |
|  | 1500 | 24.23 | 0.17 | 3.55 | 1.57 | 212.77 | 796.35 | 518.7 | 627.7 | 213.0 |
|  | 1000 | 23.12 | 0.26 | 3.66 | 1.51 | 212.77 | 796.35 | 416.8 | 569.7 | 206.1 |
|  | 600 | 21.57 | 0.43 | 3.57 | 1.47 | 212.77 | 796.35 | 356.7 | 624.7 | 213.4 |
|  | 400 | 19.20 | 0.55 | 3.37 | 1.45 | 212.77 | 796.35 | 341.5 | 765.7 | 227.9 |
|  | 200 | 12.15 | 0.67 | 2.34 | 1.44 | 212.77 | 796.35 | 315.9 | 954.8 | 242.2 |
|  | 0 | -1.37 | - | -0.30 | 1.41 | 230.20 | 926.46 | 0.0 | 0.0 | - |
| *Deficit irrigation* | | | | | | | | | | |
| NM-3 | 2000 | 26.64 | 0.13 | 2.15 | 1.62 | 242.66 | 867.67 | 721.5 | 826.2 | 264.3 |
|  | 1500 | 26.92 | 0.17 | 2.47 | 1.60 | 242.66 | 867.67 | 617.6 | 746.5 | 254.7 |
|  | 1000 | 25.34 | 0.27 | 2.63 | 1.50 | 242.66 | 867.67 | 502.0 | 690.0 | 247.1 |
|  | 600 | 22.77 | 0.43 | 2.62 | 1.43 | 242.66 | 867.67 | 400.7 | 708.9 | 250.3 |
|  | 400 | 19.77 | 0.56 | 2.58 | 1.44 | 242.66 | 867.67 | 351.4 | 802.1 | 262.2 |
|  | 200 | 11.42 | 0.66 | 1.82 | 1.46 | 242.66 | 867.67 | 306.5 | 906.0 | 273.1 |
|  | 0 | -1.77 | - | -0.48 | 1.41 | 258.61 | 948.73 | 0.0 | 0.0 | - |
| NM-5 | 2000 | 31.53 | 0.16 | 1.89 | 1.43 | 194.88 | 637.52 | 449.5 | 537.8 | 206.8 |
|  | 1500 | 31.75 | 0.21 | 1.91 | 1.50 | 194.88 | 637.52 | 442.4 | 562.2 | 212.4 |
|  | 1000 | 29.99 | 0.31 | 1.90 | 1.40 | 194.88 | 637.52 | 393.1 | 574.2 | 214.5 |
|  | 600 | 26.88 | 0.47 | 1.83 | 1.33 | 194.88 | 637.52 | 343.0 | 650.0 | 224.2 |
|  | 400 | 22.96 | 0.59 | 1.70 | 1.28 | 194.88 | 637.52 | 320.9 | 779.0 | 238.5 |
|  | 200 | 13.90 | 0.69 | 1.12 | 1.28 | 194.88 | 637.52 | 298.1 | 956.7 | 253.8 |
|  | 0 | -1.99 | - | -0.18 | 1.29 | 212.31 | 748.37 | 0.0 | 0.0 | - |
| NM-6 | 2000 | 27.63 | 0.13 | 2.29 | 1.28 | 203.00 | 757.68 | 572.5 | 657.0 | 215.0 |
|  | 1500 | 27.33 | 0.18 | 2.35 | 1.67 | 203.00 | 757.68 | 522.6 | 636.7 | 212.4 |
|  | 1000 | 25.27 | 0.26 | 2.32 | 1.57 | 203.00 | 757.68 | 449.0 | 608.0 | 208.7 |
|  | 600 | 22.86 | 0.41 | 2.28 | 1.49 | 203.00 | 757.68 | 374.5 | 644.6 | 212.8 |
|  | 400 | 20.45 | 0.55 | 2.19 | 1.44 | 203.00 | 757.68 | 338.7 | 756.5 | 224.0 |
|  | 200 | 13.32 | 0.68 | 1.54 | 1.43 | 203.00 | 757.68 | 307.1 | 947.3 | 239.0 |
|  | 0 | -1.22 | - | -0.16 | 1.44 | 226.80 | 843.75 | 0.0 | 0.0 | - |
| NM-23 | 2000 | 26.43 | 0.13 | 2.98 | 1.44 | 211.99 | 802.54 | 615.8 | 707.4 | 223.9 |
|  | 1500 | 24.46 | 0.18 | 3.29 | 1.93 | 211.99 | 802.54 | 511.0 | 622.7 | 214.2 |
|  | 1000 | 23.42 | 0.28 | 3.42 | 1.85 | 211.99 | 802.54 | 432.0 | 601.6 | 212.6 |
|  | 600 | 21.71 | 0.45 | 3.57 | 1.77 | 211.99 | 802.54 | 386.6 | 705.3 | 225.6 |
|  | 400 | 18.92 | 0.58 | 3.54 | 1.71 | 211.99 | 802.54 | 369.0 | 884.9 | 241.7 |
|  | 200 | 12.30 | 0.69 | 2.53 | 1.68 | 211.99 | 802.54 | 317.8 | 1009.9 | 250.1 |
|  | 0 | -1.56 | - | -0.31 | 1.63 | 215.81 | 923.67 | 0.0 | 0.0 | - |
| NM-69 | 2000 | 21.42 | 0.10 | 4.14 | 1.57 | 203.22 | 727.65 | 584.3 | 647.8 | 215.2 |
|  | 1500 | 19.71 | 0.19 | 4.35 | 1.90 | 203.22 | 727.65 | 405.0 | 501.4 | 195.5 |
|  | 1000 | 18.69 | 0.30 | 4.43 | 1.81 | 203.22 | 727.65 | 353.7 | 504.0 | 196.3 |
|  | 600 | 17.75 | 0.46 | 4.64 | 1.70 | 203.22 | 727.65 | 332.4 | 621.4 | 212.5 |
|  | 400 | 16.52 | 0.59 | 4.35 | 1.61 | 203.22 | 727.65 | 342.4 | 828.9 | 232.5 |
|  | 200 | 11.58 | 0.69 | 3.17 | 1.57 | 203.22 | 727.65 | 323.9 | 1030.5 | 245.9 |
|  | 0 | -1.48 | - | -0.41 | 1.52 | 233.91 | 833.39 | 0.0 | 0.0 | - |
| NM-70 | 2000 | 16.23 | 0.08 | 3.08 | 1.47 | 203.33 | 741.98 | 601.7 | 661.9 | 215.3 |
|  | 1500 | 16.11 | 0.17 | 3.22 | 1.70 | 203.33 | 741.98 | 412.7 | 501.7 | 191.7 |
|  | 1000 | 17.89 | 0.30 | 3.55 | 1.61 | 203.33 | 741.98 | 345.8 | 491.5 | 191.8 |
|  | 600 | 19.91 | 0.49 | 3.60 | 1.55 | 203.33 | 741.98 | 317.8 | 616.7 | 211.8 |
|  | 400 | 19.35 | 0.60 | 3.35 | 1.48 | 203.33 | 741.98 | 312.8 | 782.0 | 228.5 |
|  | 200 | 12.19 | 0.68 | 2.11 | 1.45 | 203.33 | 741.98 | 300.1 | 941.9 | 240.5 |
|  | 0 | -1.31 | - | -0.23 | 1.43 | 228.41 | 862.84 | 0.0 | 0.0 | - |
| NM-74 | 2000 | 32.05 | 0.18 | 3.17 | 1.43 | 229.99 | 896.35 | 641.3 | 786.5 | 241.9 |
|  | 1500 | 28.35 | 0.24 | 3.42 | 1.61 | 229.99 | 896.35 | 557.0 | 730.8 | 236.4 |
|  | 1000 | 24.13 | 0.35 | 3.61 | 1.53 | 229.99 | 896.35 | 447.1 | 686.9 | 231.5 |
|  | 600 | 19.54 | 0.51 | 3.67 | 1.49 | 229.99 | 896.35 | 348.1 | 716.0 | 234.7 |
|  | 400 | 14.35 | 0.60 | 3.39 | 1.47 | 229.99 | 896.35 | 308.5 | 783.5 | 241.4 |
|  | 200 | 7.20 | 0.68 | 2.40 | 1.47 | 229.99 | 896.35 | 292.7 | 926.2 | 254.4 |
|  | 0 | -1.66 | - | -0.75 | 1.46 | 242.31 | 975.91 | 0.0 | 0.0 | - |
| NM-77 | 2000 | 28.78 | 0.14 | 3.15 | 1.43 | 207.88 | 749.62 | 568.3 | 659.1 | 219.9 |
|  | 1500 | 29.78 | 0.21 | 3.40 | 1.74 | 207.88 | 749.62 | 492.1 | 621.0 | 215.5 |
|  | 1000 | 27.95 | 0.31 | 3.41 | 1.65 | 207.88 | 749.62 | 445.0 | 641.8 | 218.1 |
|  | 600 | 25.02 | 0.47 | 3.39 | 1.58 | 207.88 | 749.62 | 393.9 | 739.1 | 228.3 |
|  | 400 | 21.97 | 0.59 | 3.32 | 1.52 | 207.88 | 749.62 | 365.5 | 893.7 | 241.2 |
|  | 200 | 13.20 | 0.69 | 2.31 | 1.51 | 207.88 | 749.62 | 321.9 | 1027.5 | 250.0 |
|  | 0 | -1.65 | - | -0.32 | 1.48 | 234.90 | 852.51 | 0.0 | 0.0 | - |
| V-C | 2000 | 23.34 | 0.13 | 2.47 | 1.46 | 207.55 | 850.78 | 642.7 | 740.7 | 219.5 |
|  | 1500 | 24.67 | 0.17 | 2.72 | 1.70 | 207.55 | 850.78 | 593.6 | 720.2 | 218.7 |
|  | 1000 | 23.26 | 0.26 | 2.69 | 1.62 | 207.55 | 850.78 | 511.1 | 695.0 | 216.7 |
|  | 600 | 20.85 | 0.41 | 2.53 | 1.56 | 207.55 | 850.78 | 420.7 | 719.5 | 219.5 |
|  | 400 | 18.10 | 0.53 | 2.26 | 1.52 | 207.55 | 850.78 | 365.8 | 784.9 | 225.4 |
|  | 200 | 10.97 | 0.63 | 1.43 | 1.48 | 207.55 | 850.78 | 313.0 | 867.2 | 232.0 |
|  | 0 | -1.59 | - | -0.22 | 1.45 | 210.81 | 962.21 | 0.0 | 0.0 | - |
| C-76-16 | 2000 | 8.88 | 0.05 | 3.98 | 1.43 | 171.55 | 546.82 | 417.3 | 446.2 | 183.5 |
|  | 1500 | 8.74 | 0.10 | 4.06 | 1.82 | 171.55 | 546.82 | 361.3 | 406.1 | 177.5 |
|  | 1000 | 8.74 | 0.15 | 4.01 | 1.74 | 171.55 | 546.82 | 323.6 | 389.3 | 175.2 |
|  | 600 | 8.92 | 0.25 | 4.28 | 1.67 | 171.55 | 546.82 | 292.3 | 409.0 | 178.5 |
|  | 400 | 9.20 | 0.38 | 4.29 | 1.62 | 171.55 | 546.82 | 283.1 | 485.0 | 191.4 |
|  | 200 | 8.45 | 0.57 | 2.94 | 1.60 | 171.55 | 546.82 | 295.1 | 706.0 | 222.5 |
|  | 0 | -0.96 | - | -0.23 | 1.57 | 183.60 | 658.09 | 0.0 | 0.0 | - |

Supplementary table 2b: Photosynthetic process, water use efficiency and vapor pressure deficit among peanut genotypes at 70 days after sowing under full and deficit irrigated condition

| Genotype | PPFD | A  (µ mol m^-2^ s^-1^) | ΦPSII | WUE  (µmol CO_2_/m  mol H_2_O) | VPD leaf (kpa) | Fo | Fm | Fs | Fm' | Fo' |
| --- | --- | --- | --- | --- | --- | --- | --- | --- | --- | --- |
| *Full irrigation* | | | | | | | | | | |
| NM-3 | 2000 | 20.32 | 0.094 | 1.97 | 1.74 | 280.88 | 804.25 | 598.7 | 663.6 | 269.9 |
|  | 1500 | 22.46 | 0.154 | 2.36 | 1.64 | 280.88 | 804.25 | 487.7 | 577.3 | 254.3 |
|  | 1000 | 21.49 | 0.234 | 2.57 | 1.58 | 280.88 | 804.25 | 431.5 | 564.1 | 248.5 |
|  | 600 | 19.06 | 0.367 | 2.71 | 1.54 | 280.88 | 804.25 | 402.6 | 636.8 | 259.7 |
|  | 400 | 15.60 | 0.466 | 2.76 | 1.54 | 280.88 | 804.25 | 384.8 | 726.3 | 276.1 |
|  | 200 | 8.66 | 0.561 | 2.00 | 1.53 | 280.88 | 804.25 | 351.3 | 815.1 | 289.5 |
|  | 0 | -2.39 | - | -1.12 | 1.50 | 301.31 | 858.79 | 0.0 | 0.0 | - |
| NM-5 | 2000 | 33.60 | 0.167 | 2.06 | 1.55 | 232.71 | 971.00 | 678.8 | 804.2 | 220.8 |
|  | 1500 | 31.90 | 0.222 | 2.13 | 1.49 | 232.71 | 971.00 | 558.8 | 711.2 | 213.9 |
|  | 1000 | 30.08 | 0.325 | 2.18 | 1.44 | 232.71 | 971.00 | 450.1 | 667.9 | 209.7 |
|  | 600 | 25.89 | 0.480 | 2.05 | 1.43 | 232.71 | 971.00 | 361.9 | 709.9 | 212.4 |
|  | 400 | 21.33 | 0.595 | 1.98 | 1.43 | 232.71 | 971.00 | 314.9 | 790.9 | 218.8 |
|  | 200 | 11.24 | 0.672 | 1.38 | 1.46 | 232.71 | 971.00 | 286.2 | 879.5 | 225.7 |
|  | 0 | -2.92 | - | -0.47 | 1.47 | 227.70 | 895.49 | 0.0 | 0.0 | - |
| NM-6 | 2000 | 29.76 | 0.130 | 2.61 | 1.73 | 303.22 | 894.25 | 707.3 | 813.6 | 295.4 |
|  | 1500 | 29.47 | 0.195 | 2.76 | 1.63 | 303.22 | 894.25 | 558.3 | 694.4 | 276.7 |
|  | 1000 | 27.99 | 0.293 | 2.81 | 1.55 | 303.22 | 894.25 | 454.6 | 644.8 | 266.7 |
|  | 600 | 25.42 | 0.461 | 2.72 | 1.50 | 303.22 | 894.25 | 376.7 | 704.2 | 275.0 |
|  | 400 | 21.99 | 0.584 | 2.54 | 1.47 | 303.22 | 894.25 | 342.5 | 829.2 | 293.9 |
|  | 200 | 13.35 | 0.680 | 1.78 | 1.45 | 303.22 | 894.25 | 314.5 | 982.1 | 317.8 |
|  | 0 | -1.69 | - | -0.28 | 1.45 | 249.31 | 1025.79 | 0.0 | 0.0 | - |
| NM-23 | 2000 | 28.78 | 0.129 | 2.36 | 1.60 | 226.42 | 906.70 | 799.1 | 917.7 | 227.2 |
|  | 1500 | 28.22 | 0.177 | 2.47 | 1.52 | 226.42 | 906.70 | 686.3 | 833.9 | 221.6 |
|  | 1000 | 26.36 | 0.272 | 2.47 | 1.47 | 226.42 | 906.70 | 545.2 | 749.5 | 215.1 |
|  | 600 | 23.65 | 0.433 | 2.39 | 1.43 | 226.42 | 906.70 | 417.6 | 740.7 | 214.5 |
|  | 400 | 20.31 | 0.562 | 2.19 | 1.42 | 226.42 | 906.70 | 346.1 | 794.4 | 218.7 |
|  | 200 | 12.25 | 0.676 | 1.44 | 1.42 | 226.42 | 906.70 | 293.9 | 906.3 | 226.4 |
|  | 0 | -1.92 | - | -0.25 | 1.41 | 234.85 | 895.60 | 0.0 | 0.0 | - |
| NM-69 | 2000 | 24.75 | 0.111 | 3.03 | 1.75 | 244.31 | 985.76 | 605.3 | 684.2 | 219.3 |
|  | 1500 | 25.35 | 0.184 | 3.27 | 1.65 | 244.31 | 985.76 | 475.6 | 584.8 | 207.9 |
|  | 1000 | 24.23 | 0.287 | 3.48 | 1.58 | 244.31 | 985.76 | 405.5 | 569.9 | 206.4 |
|  | 600 | 22.31 | 0.453 | 3.42 | 1.52 | 244.31 | 985.76 | 352.9 | 645.8 | 215.9 |
|  | 400 | 19.66 | 0.582 | 3.30 | 1.51 | 244.31 | 985.76 | 330.2 | 790.5 | 230.1 |
|  | 200 | 11.99 | 0.686 | 2.29 | 1.50 | 244.31 | 985.76 | 300.6 | 956.3 | 242.5 |
|  | 0 | -1.76 | - | -0.39 | 1.47 | 232.71 | 971.00 | 0.0 | 0.0 | - |
| NM-70 | 2000 | 27.60 | 0.117 | 1.91 | 1.57 | 236.42 | 880.00 | 800.9 | 906.1 | 238.3 |
|  | 1500 | 27.28 | 0.166 | 2.09 | 1.50 | 236.42 | 880.00 | 674.6 | 808.7 | 231.0 |
|  | 1000 | 24.01 | 0.245 | 2.02 | 1.44 | 236.42 | 880.00 | 539.3 | 717.0 | 222.7 |
|  | 600 | 20.22 | 0.378 | 1.97 | 1.42 | 236.42 | 880.00 | 405.0 | 660.6 | 216.6 |
|  | 400 | 16.85 | 0.498 | 2.09 | 1.40 | 236.42 | 880.00 | 331.2 | 681.5 | 217.9 |
|  | 200 | 9.43 | 0.634 | 1.72 | 1.40 | 236.42 | 880.00 | 300.1 | 836.3 | 232.3 |
|  | 0 | -1.83 | - | -0.78 | 1.40 | 239.68 | 896.73 | 0.0 | 0.0 | - |
| NM-74 | 2000 | 33.65 | 0.172 | 2.21 | 1.80 | 250.43 | 934.87 | 553.0 | 673.2 | 222.2 |
|  | 1500 | 32.32 | 0.228 | 2.38 | 1.71 | 250.43 | 934.87 | 489.2 | 640.7 | 218.5 |
|  | 1000 | 29.85 | 0.334 | 2.57 | 1.63 | 250.43 | 934.87 | 418.8 | 640.2 | 219.1 |
|  | 600 | 24.89 | 0.489 | 2.79 | 1.60 | 250.43 | 934.87 | 349.9 | 703.0 | 226.9 |
|  | 400 | 16.84 | 0.569 | 3.32 | 1.62 | 250.43 | 934.87 | 309.3 | 750.3 | 230.7 |
|  | 200 | 7.38 | 0.672 | 2.23 | 1.61 | 250.43 | 934.87 | 298.4 | 918.1 | 248.6 |
|  | 0 | -2.22 | - | -1.42 | 1.59 | 226.47 | 903.97 | 0.0 | 0.0 | - |
| NM-77 | 2000 | 33.26 | 0.157 | 2.34 | 1.62 | 239.39 | 758.84 | 630.8 | 749.7 | 237.0 |
|  | 1500 | 32.55 | 0.216 | 2.46 | 1.51 | 239.39 | 758.84 | 544.5 | 696.3 | 231.3 |
|  | 1000 | 29.13 | 0.310 | 2.30 | 1.45 | 239.39 | 758.84 | 471.5 | 688.4 | 231.3 |
|  | 600 | 26.52 | 0.479 | 2.27 | 1.40 | 239.39 | 758.84 | 378.3 | 731.2 | 236.8 |
|  | 400 | 22.82 | 0.595 | 2.13 | 1.37 | 239.39 | 758.84 | 341.6 | 848.4 | 248.4 |
|  | 200 | 12.69 | 0.674 | 1.33 | 1.37 | 239.39 | 758.84 | 310.6 | 953.5 | 256.8 |
|  | 0 | -2.08 | - | -0.23 | 1.37 | 241.93 | 962.85 | 0.0 | 0.0 | - |
| V-C | 2000 | 22.40 | 0.117 | 1.85 | 1.63 | 257.19 | 929.39 | 562.4 | 637.1 | 222.9 |
|  | 1500 | 24.06 | 0.151 | 2.08 | 1.53 | 257.19 | 929.39 | 524.6 | 619.1 | 223.2 |
|  | 1000 | 22.54 | 0.224 | 2.09 | 1.47 | 257.19 | 929.39 | 457.0 | 590.0 | 220.5 |
|  | 600 | 19.76 | 0.357 | 1.96 | 1.42 | 257.19 | 929.39 | 401.3 | 625.6 | 225.7 |
|  | 400 | 16.40 | 0.465 | 1.75 | 1.41 | 257.19 | 929.39 | 372.8 | 703.5 | 234.7 |
|  | 200 | 9.15 | 0.568 | 1.05 | 1.38 | 257.19 | 929.39 | 343.2 | 805.7 | 245.0 |
|  | 0 | -2.29 | - | -0.25 | 1.36 | 280.88 | 804.25 | 0.0 | 0.0 | - |
| C-76-16 | 2000 | 27.83 | 0.122 | 3.15 | 1.69 | 241.93 | 962.85 | 686.5 | 786.4 | 228.2 |
|  | 1500 | 27.25 | 0.186 | 3.51 | 1.60 | 241.93 | 962.85 | 549.2 | 679.0 | 217.7 |
|  | 1000 | 25.60 | 0.284 | 3.61 | 1.54 | 241.93 | 962.85 | 432.2 | 608.3 | 210.2 |
|  | 600 | 23.23 | 0.447 | 3.48 | 1.50 | 241.93 | 962.85 | 356.1 | 646.6 | 215.2 |
|  | 400 | 20.25 | 0.568 | 3.25 | 1.48 | 241.93 | 962.85 | 334.2 | 774.7 | 228.0 |
|  | 200 | 12.24 | 0.674 | 2.24 | 1.47 | 241.93 | 962.85 | 309.8 | 949.1 | 241.1 |
|  | 0 | -1.81 | - | -0.30 | 1.45 | 244.51 | 943.37 | 0.0 | 0.0 | - |
| *Deficit irrigation* | | | | | | | | | | |
| NM-3 | 2000 | 31.13 | 0.146 | 2.42 | 1.63 | 258.55 | 1016.21 | 732.6 | 858.1 | 247.2 |
|  | 1500 | 30.39 | 0.189 | 2.58 | 1.53 | 258.55 | 1016.21 | 627.3 | 773.8 | 239.7 |
|  | 1000 | 28.45 | 0.289 | 2.65 | 1.47 | 258.55 | 1016.21 | 509.5 | 716.6 | 233.9 |
|  | 600 | 25.67 | 0.459 | 2.64 | 1.47 | 258.55 | 1016.21 | 401.8 | 743.6 | 236.7 |
|  | 400 | 22.18 | 0.592 | 2.58 | 1.49 | 258.55 | 1016.21 | 349.8 | 858.0 | 247.1 |
|  | 200 | 12.59 | 0.676 | 1.80 | 1.44 | 258.55 | 1016.21 | 309.1 | 955.6 | 254.6 |
|  | 0 | -1.88 | - | -0.42 | 1.46 | 240.80 | 946.53 | 0.0 | 0.0 | - |
| NM-5 | 2000 | 33.14 | 0.158 | 1.84 | 1.54 | 247.68 | 923.47 | 590.6 | 702.9 | 220.7 |
|  | 1500 | 32.15 | 0.199 | 1.81 | 1.43 | 247.68 | 923.47 | 564.3 | 705.7 | 223.6 |
|  | 1000 | 30.21 | 0.300 | 1.79 | 1.36 | 247.68 | 923.47 | 478.8 | 685.1 | 222.9 |
|  | 600 | 27.06 | 0.460 | 1.70 | 1.31 | 247.68 | 923.47 | 386.3 | 717.7 | 228.0 |
|  | 400 | 23.54 | 0.588 | 1.55 | 1.31 | 247.68 | 923.47 | 339.6 | 827.0 | 239.3 |
|  | 200 | 14.17 | 0.693 | 0.98 | 1.32 | 247.68 | 923.47 | 305.8 | 997.2 | 252.6 |
|  | 0 | -2.08 | - | -0.15 | 1.32 | 263.42 | 1048.11 | 0.0 | 0.0 | - |
| NM-6 | 2000 | 27.05 | 0.142 | 2.66 | 1.70 | 233.83 | 964.73 | 529.3 | 617.4 | 205.8 |
|  | 1500 | 26.09 | 0.181 | 2.87 | 1.60 | 233.83 | 964.73 | 469.7 | 572.5 | 200.0 |
|  | 1000 | 23.50 | 0.268 | 3.05 | 1.52 | 233.83 | 964.73 | 408.2 | 556.3 | 198.1 |
|  | 600 | 21.78 | 0.424 | 3.13 | 1.47 | 233.83 | 964.73 | 359.6 | 624.5 | 206.5 |
|  | 400 | 19.92 | 0.552 | 2.87 | 1.46 | 233.83 | 964.73 | 341.6 | 764.7 | 219.9 |
|  | 200 | 13.07 | 0.679 | 2.05 | 1.47 | 233.83 | 964.73 | 314.4 | 979.2 | 234.7 |
|  | 0 | -1.33 | - | -0.26 | 1.47 | 258.55 | 1016.21 | 0.0 | 0.0 | - |
| NM-23 | 2000 | 25.44 | 0.128 | 39.07 | 1.96 | 257.60 | 1039.72 | 423.9 | 472.9 | 192.8 |
|  | 1500 | 22.91 | 0.176 | 25.42 | 1.88 | 257.60 | 1039.72 | 365.2 | 427.2 | 185.8 |
|  | 1000 | 22.01 | 0.278 | 20.31 | 1.80 | 257.60 | 1039.72 | 328.8 | 426.2 | 185.5 |
|  | 600 | 20.63 | 0.449 | 20.87 | 1.74 | 257.60 | 1039.72 | 308.9 | 499.3 | 195.7 |
|  | 400 | 18.16 | 0.584 | 18.91 | 1.71 | 257.60 | 1039.72 | 307.7 | 637.1 | 212.7 |
|  | 200 | 12.27 | 0.687 | 12.65 | 1.66 | 257.60 | 1039.72 | 307.6 | 864.6 | 243.0 |
|  | 0 | -1.48 | - | -1.25 | 1.60 | 242.39 | 954.29 | 0.0 | 0.0 | - |
| NM-69 | 2000 | 19.31 | 0.082 | 15.98 | 1.94 | 240.80 | 946.53 | 429.8 | 467.7 | 185.8 |
|  | 1500 | 17.02 | 0.188 | 11.77 | 1.84 | 240.80 | 946.53 | 317.6 | 379.5 | 172.4 |
|  | 1000 | 16.20 | 0.295 | 10.44 | 1.74 | 240.80 | 946.53 | 288.7 | 389.8 | 174.6 |
|  | 600 | 15.74 | 0.462 | 11.37 | 1.65 | 240.80 | 946.53 | 283.8 | 490.8 | 192.1 |
|  | 400 | 15.11 | 0.584 | 10.97 | 1.60 | 240.80 | 946.53 | 309.7 | 700.9 | 219.3 |
|  | 200 | 11.35 | 0.685 | 7.83 | 1.55 | 240.80 | 946.53 | 309.3 | 943.1 | 240.0 |
|  | 0 | -1.35 | - | -0.81 | 1.51 | 231.99 | 982.58 | 0.0 | 0.0 | - |
| NM-70 | 2000 | 24.93 | 0.122 | 4.35 | 1.73 | 248.41 | 910.46 | 665.7 | 770.1 | 234.7 |
|  | 1500 | 23.10 | 0.195 | 4.21 | 1.65 | 248.41 | 910.46 | 516.1 | 649.2 | 217.8 |
|  | 1000 | 23.21 | 0.309 | 4.44 | 1.58 | 248.41 | 910.46 | 424.5 | 617.1 | 215.7 |
|  | 600 | 23.51 | 0.493 | 4.00 | 1.51 | 248.41 | 910.46 | 350.8 | 689.9 | 228.1 |
|  | 400 | 21.82 | 0.610 | 3.46 | 1.48 | 248.41 | 910.46 | 324.3 | 830.4 | 242.0 |
|  | 200 | 13.49 | 0.689 | 2.12 | 1.47 | 248.41 | 910.46 | 306.6 | 984.6 | 253.6 |
|  | 0 | -1.52 | - | -0.22 | 1.46 | 245.62 | 1001.20 | 0.0 | 0.0 | - |
| NM-74 | 2000 | 32.06 | 0.172 | 3.14 | 1.65 | 241.52 | 922.16 | 717.0 | 865.3 | 239.3 |
|  | 1500 | 28.80 | 0.221 | 3.31 | 1.57 | 241.52 | 922.16 | 632.2 | 810.0 | 234.6 |
|  | 1000 | 25.29 | 0.321 | 3.47 | 1.52 | 241.52 | 922.16 | 518.4 | 760.9 | 230.0 |
|  | 600 | 21.50 | 0.486 | 3.58 | 1.50 | 241.52 | 922.16 | 400.3 | 775.0 | 231.4 |
|  | 400 | 17.42 | 0.597 | 3.35 | 1.50 | 241.52 | 922.16 | 336.4 | 833.6 | 236.7 |
|  | 200 | 9.72 | 0.684 | 2.57 | 1.49 | 241.52 | 922.16 | 298.6 | 946.3 | 245.7 |
|  | 0 | -1.87 | - | -0.77 | 1.46 | 230.81 | 930.93 | 0.0 | 0.0 | - |
| NM-77 | 2000 | 26.70 | 0.129 | 2.74 | 1.77 | 249.28 | 965.49 | 671.3 | 770.1 | 233.3 |
|  | 1500 | 29.05 | 0.198 | 3.09 | 1.68 | 249.28 | 965.49 | 633.6 | 788.4 | 234.0 |
|  | 1000 | 27.90 | 0.296 | 3.21 | 1.61 | 249.28 | 965.49 | 557.3 | 789.0 | 234.6 |
|  | 600 | 24.89 | 0.456 | 3.22 | 1.55 | 249.28 | 965.49 | 461.7 | 846.7 | 240.3 |
|  | 400 | 21.82 | 0.588 | 3.20 | 1.54 | 249.28 | 965.49 | 393.2 | 953.1 | 248.4 |
|  | 200 | 13.49 | 0.690 | 2.34 | 1.52 | 249.28 | 965.49 | 326.7 | 1055.3 | 254.8 |
|  | 0 | -1.75 | - | -0.33 | 1.50 | 249.30 | 1046.58 | 0.0 | 0.0 | - |
| V-C | 2000 | 23.74 | 0.120 | 2.56 | 1.73 | 244.80 | 950.92 | 729.3 | 828.5 | 235.7 |
|  | 1500 | 24.10 | 0.167 | 2.75 | 1.66 | 244.80 | 950.92 | 633.3 | 760.0 | 229.5 |
|  | 1000 | 23.01 | 0.253 | 2.78 | 1.59 | 244.80 | 950.92 | 526.0 | 705.4 | 223.8 |
|  | 600 | 21.14 | 0.411 | 2.77 | 1.55 | 244.80 | 950.92 | 427.4 | 726.9 | 226.0 |
|  | 400 | 19.09 | 0.542 | 2.66 | 1.51 | 244.80 | 950.92 | 373.4 | 815.3 | 234.3 |
|  | 200 | 12.16 | 0.666 | 1.81 | 1.48 | 244.80 | 950.92 | 308.9 | 924.2 | 242.8 |
|  | 0 | -1.46 | - | -0.23 | 1.46 | 242.48 | 912.43 | 0.0 | 0.0 | - |
| C-76-16 | 2000 | 16.83 | 0.105 | 6.28 | 1.85 | 250.35 | 1050.44 | 434.6 | 496.7 | 193.3 |
|  | 1500 | 16.06 | 0.155 | 5.18 | 1.77 | 250.35 | 1050.44 | 382.5 | 465.9 | 188.6 |
|  | 1000 | 15.15 | 0.231 | 4.76 | 1.70 | 250.35 | 1050.44 | 342.8 | 467.4 | 188.6 |
|  | 600 | 13.91 | 0.352 | 5.38 | 1.65 | 250.35 | 1050.44 | 320.4 | 544.4 | 197.4 |
|  | 400 | 12.67 | 0.466 | 5.30 | 1.64 | 250.35 | 1050.44 | 314.0 | 658.5 | 210.4 |
|  | 200 | 9.41 | 0.611 | 3.23 | 1.61 | 250.35 | 1050.44 | 310.3 | 838.3 | 232.7 |
|  | 0 | -1.17 | - | -0.31 | 1.58 | 251.02 | 880.17 | 0.0 | 0.0 | - |
